# Astrocyte and Microglial Transcriptional Programs in the Atypical Teratoid/Rhabdoid Tumor Microenvironment

**DOI:** 10.3390/cancers18213455

**Published:** 2026-10-26

**Authors:** Yu Xue, Uvindu Thilanka, Drishty Badhon Sarker, Md Al Azad, Qing-Xiang Amy Sang

**Affiliations:** 1 Chemistry and Biochemistry, Florida State University, Tallahassee, FL 32306, USA; yx21@fsu.edu (Y.X.); ud24@fsu.edu (U.T.); ds22@fsu.edu (D.B.S.); ma25h@fsu.edu (M.A.A.); 2 Institute of Molecular Biophysics, Florida State University, Tallahassee, FL 32306, USA; 3 Institute for Pediatric Rare Diseases, Florida State University, Tallahassee, FL 32306, USA

**Keywords:** atypical teratoid/rhabdoid tumor (ATRT), tumor microenvironment, astrocytes, microglia, single-nucleus RNA sequencing, transcriptional programs, CellChat, glia–tumor interactions

## Abstract

**Background/Objectives**: The original GSE283839 study characterized malignant-cell heterogeneity in atypical teratoid/rhabdoid tumor (ATRT). Building on this resource, we investigated the less-characterized glial microenvironment, focusing on astrocyte and microglial transcriptional programs and expression-supported candidate interactions with tumor cells. **Methods**: We reanalyzed single-nucleus RNA-sequencing data from 12 ATRT tumors using published annotations, program scoring, paired astrocyte–tumor comparisons, and conservative annotation sensitivities. CellChat analyses used 10,000 permutations and within-model Benjamini–Hochberg (BH) correction. An independent cohort comprised eight specimens from four patients. **Results**: The discovery dataset included 47 annotated astrocytes and 288 annotated microglia. Astrocytes expressed *SLC1A2*, *SLC1A3*, and *GLUL*, with higher glutamate/glutamine-module scores than matched tumor cells in two of three evaluable tumors. This difference persisted in the single tumor evaluable under conservative annotation. Microglia exhibited overlapping inflammatory, immunoregulatory, phagocytic/lipid-processing, and antigen-presentation programs. Across 12 tumors, microglial *CD74* expression correlated with the antigen-presentation module score. Candidate interactions included astrocyte-to-tumor *NCAM1–FGFR1* and *PTN–PTPRZ1* and microglia-to-tumor *SPP1*-related pairs. Tumor-to-microglia *APP–CD74* passed within-model BH correction in 7/10 eligible discovery tumors. External analyses supported tumor-to-myeloid *APP–CD74* in at least one myeloid population in each of five evaluable specimens from four patients. **Conclusions**: This exploratory study highlights astrocyte metabolic expression programs and immune-related microglial programs in ATRT, prioritizing *APP–CD74* and additional expression-supported glia–tumor interaction candidates for future spatial and functional investigation.

## Simple Summary

This re-analysis of published snRNA-seq data focuses on the transcriptional programs and putative functions of the nonmalignant compartment in 12 atypical teratoid/rhabdoid tumors (ATRT). Using the original annotations, only 516 (2.94%) of the 17,564 recovered nuclei were classified as nonmalignant, with microglia accounting for more than half (55.81%) of this population. Despite their low abundance, astrocytes emerged as a distinct and heterogeneous component of the tumor microenvironment (TME). Astrocytes consistently expressed genes associated with glutamate–glutamine metabolism, including *SLC1A3*, *SLC1A2*, and *GLUL*, supporting a potential metabolic role within the TME. They also expressed growth- and neurodevelopment-associated factors such as *NCAM1*, *PTN*, *PDGFC*, and *FGF2*. Microglia displayed overlapping homeostatic, inflammatory, immunoregulatory, phagocytic, antigen-presentation, and stress-response programs, highlighting diverse functional states. Cell–cell interaction analyses identified candidate astrocyte–tumor interactions (*NCAM1–FGFR1* and *PTN–PTPRZ1*) and microglia–tumor interactions (*APP–CD74* and *SPP1*-related pathways). Among these, *APP–CD74* showed consistent support across external specimens, prioritizing it for future functional investigation.

## 1. Introduction

Atypical teratoid/rhabdoid tumor (ATRT) is an aggressive central nervous system malignancy that occurs predominantly in infants and young children. ATRT is characterized by loss of *SMARCB1*/INI1, or less commonly *SMARCA4*/BRG1, core components of the SWI/SNF chromatin-remodeling complex [1]. Despite multimodal treatment, the disease remains clinically challenging because of its early occurrence, rapid progression, and the toxicities associated with intensive therapy [1]. EU-RHAB registry data further underscore the clinical vulnerability of infants and newborns with ATRT, for whom age, dissemination or synchronous disease, and treatment intensity constrain management [1]. Molecular profiling has resolved ATRT into the ATRT-MYC, ATRT-SHH, and ATRT-TYR groups, which differ in epigenetic state, transcriptional programs, developmental context, and clinical associations [2,3,4,5]. These tumor-intrinsic differences provide an essential context for interpreting ATRT biology, but they do not fully explain how nonmalignant cells within the tumor niche may influence malignant cell states, treatment responses, and disease progression.

The ATRT tumor microenvironment (TME) remains less well understood than the malignant compartment. The study associated with GSE283839 focused on malignant cell-state organization and identified a cycling progenitor-like population at the base of ATRT differentiation trajectories [2]. Prior ATRT studies have shown subgroup-dependent lymphoid and myeloid infiltration, macrophage–tumor effects, ecosystem responses to nutrient stress, and microenvironmental changes during progression [6,7,8,9]. Comparative analyses of pediatric brain tumors further support disease-specific interpretation of immune and stromal populations [10]. In the deposited GSE283839 subset, source-labeled microglia were the largest recovered nonmalignant group, while astrocytes were sparse. These source labels provide a starting point for a focused analysis, but their correspondence to resident microglia or other macrophage populations requires additional assessment.

Astrocytes maintain neural tissue homeostasis through neurotransmitter clearance, metabolic buffering, ion and water balance, trophic support, extracellular-matrix organization, and interactions with vascular and immune cells [11,12,13,14,15]. Their transcriptional responses to injury and disease are heterogeneous and context-dependent; experimental and consensus frameworks discourage reducing this diversity to a single “reactive” identity or a fixed binary nomenclature [11,16,17]. In brain tumors, astrocytes may influence malignant phenotypes through soluble factors, extracellular matrix, direct contact, and metabolite exchange. Prior experimental work in other tumor systems has shown that astrocyte coculture can alter the treatment response of glioblastoma (GBM) stem-like cells [18], while glutamine synthetase activity can sustain nucleotide biosynthesis under glutamine restriction [19]. Astrocyte–glioma metabolic coupling can also have context-dependent consequences, including glutamate clearance and glutamine availability [20]. More recently, direct mitochondrial transfer from astrocytes was shown to enhance glioblastoma tumorigenicity in an experimental model [21]. These studies provide testable interpretive foundations for expression patterns in ATRT-associated astrocytes.

Microglia are resident central nervous system myeloid cells, whereas other tumor-associated macrophages can have different developmental and anatomical origins. Their expression programs form continua influenced by tissue context, hypoxia, tumor-derived cues, and therapy [22,23,24,25,26,27,28,29,30]. Inflammatory-associated and immunoregulatory-associated programs may coexist with lipid-associated, interferon-responsive, stress-associated, proliferative, or antigen-presentation-associated expression [22,23,27,28,30]. The *CXCL9*:*SPP1* framework similarly relates macrophage expression to broader multicellular programs [31]. Glioma studies have also examined *SPP1–CD44*-related biology [32,33], providing context for candidate selection rather than direct evidence in ATRT. Because many activation-associated genes are shared, *SPP1*, *APOE*, *APOC1*, *GPNMB*, or *CD163* expression alone cannot establish resident microglial origin or blood-derived infiltration. We therefore distinguish source labels, conservative identity assignments, and state-associated scores. Throughout the discovery analyses, “microglia” refers to the published Microglia label unless a conservative subset is specified. Macrophage-like, BAM-like, and unresolved myeloid populations identified by marker refinement are reported separately.

Single-cell and single-nucleus transcriptomics provide an opportunity to examine these cellular programs at a resolution that is not accessible through bulk sequencing. Beyond identifying rare nonmalignant populations, these approaches can reveal coexisting transcriptional programs and nominate candidate interactions between tumor and stromal or immune compartments. Integrating transcriptional program analysis with ligand–receptor inference can therefore generate mechanistic hypotheses regarding how astrocytes and microglia may participate in the ATRT tumor microenvironment and identify pathways for subsequent experimental investigation.

**Figure 1:**
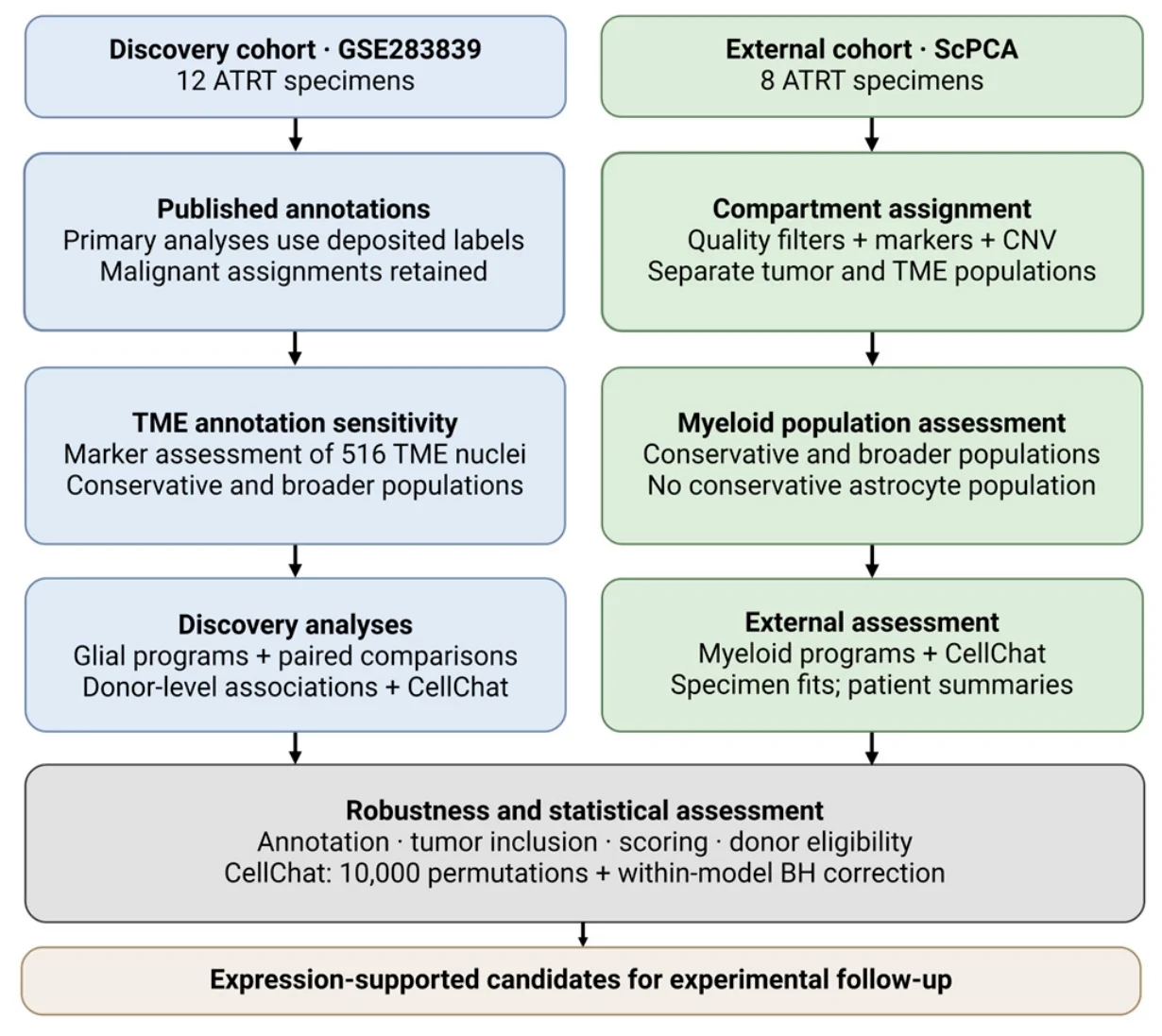
Analysis workflow for the discovery and external ATRT cohorts. The discovery cohort (GSE283839) comprised 17,564 nuclei from 12 tumors, including 516 nonmalignant tumor microenvironment (TME) nuclei. Primary analyses used published cell labels, supported by quality filtering, doublet removal, cluster composition, copy-number evidence, and reference-signature scoring (Section 2.3). All malignant assignments were retained. Annotation sensitivity analyses were restricted to TME nuclei, using composite markers, required identity genes, competing-lineage checks, and enrichment and neighborhood support to define conservative and broader populations (Section 2.11; Table 1). Discovery analyses included glial program scoring, paired astrocyte–tumor comparisons, donor-level associations, and CellChat inference with same-donor expression checks. The external ScPCA cohort (SCPCP000010) comprised eight specimens from four patients. Quality filters, marker evidence, and source inferCNV results supported compartment assignments, including a separate CNV-supported tumor population; automated annotations provided supplementary information. Here, “myeloid” encompasses microglial and macrophage-like populations. External interaction support in a specimen indicates support in at least one eligible myeloid population and does not establish a microglia-specific association. Myeloid programs and specimen-specific CellChat results were evaluated, with repeated specimens linked by patient (Section 2.12; Table S17). No astrocytes met conservative criteria, and molecular-subtype labels were unavailable. Sensitivity analyses assessed annotation, tumor inclusion, scoring, donor eligibility, and minimum population sizes. CellChat models used 10,000 permutations and within-model Benjamini–Hochberg (BH) correction. Conservative and broader populations overlap and were used as sensitivity definitions, not independent observations. Arrows indicate analysis flow toward expression-supported candidates for experimental follow-up. CNV, copy-number variation.

The contribution of this study is a focused, descriptive examination of the nonmalignant component of the published atlas, rather than a new malignant-cell taxonomy. We evaluated astrocyte and microglial programs, paired astrocyte–tumor expression, and candidate ligand–receptor associations, then tested how annotation, sample inclusion, gene scoring, tumor scope, and minimum group size influence interpretation. We additionally examined an external ScPCA cohort. This framework identifies candidate observations and assesses their sensitivity to analytical choices and available donor support while preserving every source malignant assignment in GSE283839. An overview is provided in Figure 1.

**Table 1:** Marker panels used for conservative astrocyte and myeloid annotation.

Panel	Genes	Sources
Astrocyte	*ALDH1L1*, *AQP4*, *SLC1A2*, *SLC1A3*, *GFAP*, *SOX9*	[12,13]
Astrocyte non-transporter	*ALDH1L1*, *AQP4*, *GFAP*, *SOX9*, *S100B*, *FGFR3*	[12,13]
Microglia	*P2RY12*, *TMEM119*, *GPR34*, *SALL1*, *P2RY13*, *SLC2A5*, *OLFML3*, *CX3CR1*	[24,27,28]
Myeloid lineage	*LST1*, *TYROBP*, *FCER1G*, *AIF1*, *CSF1R*, *SPI1*, *CTSS*, *CD68*, *C1QA*, *C1QB*, *C1QC*	[27,28,34]
Macrophage	*CD163*, *MRC1*, *MS4A7*, *CD14*, *LYZ*, *FCGR3A*, *ITGA4*, *F13A1*, *STAB1*	[27,28,34]
BAM-like	*MRC1*, *LYVE1*, *F13A1*, *CD163*, *STAB1*	[34]
Monocyte	*FCN1*, *VCAN*, *S100A8*, *S100A9*, *CCR2*	[27,34]

## 2. Materials and Methods

### 2.1. Data Acquisition

The publicly available GSE283839 single-nucleus RNA-sequencing dataset was initially accessed on 15 February 2026. This is the snRNA_10Xv3 subseries of GSE283843; dataset generation, sample collection, processing, annotations, and malignant-state definitions are described in the source publication [2]. The deposited files are GSE283839_ATRT_RNA_V3_metadata.tsv.gz and GSE283839_ATRT_RNA_V3_counts_raw.tsv.gz; the local metadata and counts were analyzed after decompression. On 7 October 2026, the GEO record and both downloads were rechecked, and SHA-256 checksums of the decompressed downloads matched the local analysis files exactly (Supplementary Table S9 provenance files). The GEO series was public on 24 July 2025 and listed a last update of 12 March 2026 at verification; this record-update date is distinct from the original retrieval date. The raw matrix contained 36,601 genes and 17,564 nuclei from three ATRT-MYC, five ATRT-SHH, and four ATRT-TYR tumors. Cell2Sentence outputs were matched by cell identifier for supplementary comparison only.

The analyzed cohort is the 12-tumor 10X v3 subset of the larger 19-tumor atlas. The published supplement reports snap-frozen tissue for all 12 and extended labels SHH-1A for SHH-1 through SHH-4 and SHH-2 for SHH-5; broad labels are MYC (three), SHH (five), and TYR (four). Age, tumor grade, site, treatment, and outcomes are not reported for these 12 tumors in the deposited GEO metadata or the relevant published patient table. The published per-nucleus gender field is missing for all 12; sex values for the seven other atlas tumors were not transferred to this subset. Table S14 lists source identifiers, reported tissue and technology, and explicit missing fields. The 17,564 cell identifiers and malignant/TME assignments match the published supplement exactly.

### 2.2. Count-Matrix and Metadata Processing

Cell identifiers in the count matrix, metadata, and Cell2Sentence files were reconciled using the transformations documented in the accompanying scripts. All included nuclei were matched unambiguously across the relevant files. Raw counts were analyzed only after identifier matching and subsetting to the relevant published annotations.

For astrocyte state-program scoring, genes were ranked from highest to lowest raw count within each astrocyte nucleus. Because library-size normalization rescales the counts within a nucleus without changing their relative order, it does not affect these rank-based scores. For the astrocyte marker-expression summaries, raw counts were independently normalized to counts per 10,000 (CP10K) and transformed as ln(1 + CP10K). Raw counts used in the normalized-expression microglial analyses were processed in the same manner. For paired astrocyte–tumor pseudobulk comparisons, raw counts were summed within each biological sample and cellular compartment and transformed as log_2_(CPM + 1). Cell2Sentence sensitivity analyses used ranked gene positions rather than normalized count values. The cellular-landscape analysis used the deposited cell labels and Harmony coordinates without additional filtering or re-embedding.

### 2.3. Use of Published Cell-Type Annotations

In the source study, Blanco-Carmona et al. [2] filtered nuclei by transcript and gene counts and mitochondrial RNA content, removed doublets with Scrublet (version 0.2.1) using patient-specific thresholds, and normalized expression per patient. They distinguished the TME from malignant cells by examining patient composition within expression-based clusters: clusters shared across patients were assigned to the TME, whereas clusters dominated by a single patient were considered malignant. Using TME clusters as a reference, inferCNV analysis of chromosome 22q loss provided additional support for malignant assignments in ATRT-TYR; ATRT subtype gene signatures were also examined. Harmony integration accounted for patient and sequencing-technology effects. TME identities were assigned using UCell enrichment scores (the UCell version was not reported in the source publication) for PanglaoDB marker sets. Malignant states were defined by clustering enrichment scores for human fetal-brain, recurrent pan-cancer, and choroid-plexus gene sets. The authors prioritized pan-cancer states over normal-cell signatures, discarded broadly enriched signatures, and retained the label Unannotated when no clear state signature was present [2].

This documented workflow provided a methodological basis for retaining the published classifications in our primary analyses and maintaining comparability with the source study. The source GEO Final_Annotation labels define the baseline analyses: Astrocytes (*n* = 47) and Microglia (*n* = 288). “Source-labeled microglia” denotes that deposited category and does not establish resident ontogeny or exclude macrophage-like cells. We retained the malignant-versus-nonmalignant boundary and all malignant-state labels without reannotation, preserving the source study’s distinction between malignant cells expressing neural or glial programs and nonmalignant brain lineages. Only the 516 source TME nuclei underwent marker-based refinement for sensitivity analyses (Section 2.11). Cell2Sentence was a supplementary comparison of annotation methods applied to the same expression data, not independent validation.

Cell-type predictions generated using Cell2Sentence, a large language model (LLM)-based framework for single-cell transcriptomic analysis, were compared separately with the published GEO annotations. To permit a direct comparison, the more detailed labels produced by each annotation approach were mapped to a common set of broad cell-lineage categories using the predefined mapping reported in Supplementary Table S6. Following this mapping, 376 of the 516 nuclei classified as nonmalignant tumor-microenvironment cells (72.9%) were assigned to the same broad lineage by both approaches (Supplementary Figure S1A,B; Supplementary Table S6). Because both approaches evaluated the same transcriptomic data and the published GEO annotations do not represent an independent biological reference standard, their agreement was interpreted descriptively rather than as validation of either system. Differences may reflect variation in label definitions, classification methods, or annotation granularity.

### 2.4. Tumor and Tumor Microenvironment Classification

For the dataset-wide cellular-landscape analysis, nuclei with Final_Annotation_Focus = TME were assigned to the nonmalignant tumor-microenvironment (TME) compartment, and all remaining nuclei were assigned to the tumor compartment. This rule classified 516 nuclei as TME and 17,048 as tumor among the 17,564 nuclei in the deposited cohort. The tumor compartment included 7676 nuclei labeled Unannotated, which the source workflow classified as tumor nuclei lacking a specific tumor-state signature, and 129 nuclei assigned to the Immune-like tumor state. Oligodendrocyte precursor cell (OPC) denoted a nonmalignant TME population, whereas OPC-like denoted a malignant tumor state. Overall tumor and TME percentages were calculated using all 17,564 nuclei as the denominator; percentages of individual TME populations were calculated using the 516 TME nuclei as the denominator.

Within the source TME compartment, Final_Annotation identified Microglia (*n* = 288), OPC (*n* = 79), Endothelial cells (*n* = 48), Astrocytes (*n* = 47), Neurons (*n* = 34), and Pericytes (*n* = 20). The Microglia category was the only separately resolved immune label; this does not establish a pure resident lineage. T-cell, B-cell, plasma-cell, and natural-killer-cell populations were not separately resolved in the deposited annotations. Their biological abundance was therefore not assigned as zero. No immune-hot or immune-cold designation was inferred from recovered nuclear proportions.

The state-resolved CellChat analyses included astrocytes, source-labeled microglia, and malignant cells assigned to eight published states: choroid-plexus-like (CP-like), cilia-like, hypoxic, neuronal-intermediate-precursor-cell-like (IPC-like), mesenchymal-like, neuronal-precursor-cell-like (NPC-like), oligodendrocyte-precursor-cell-like (OPC-like), and radial-glia-like (RG-like). The 7676 Unannotated tumor nuclei were excluded because they lacked a resolved malignant-state label; the 129 Immune-like tumor nuclei and other TME populations were outside that initial scope. The excluded Unannotated population accounts for 45.0% of the author-defined tumor compartment. Consequently, the initial interactome describes the selected state-resolved tumor subset and may omit ligand–receptor expression or alter averaged expression, group abundance, and inferred probabilities relative to the full tumor compartment. We therefore compared the eight malignant states with those states plus Unannotated nuclei while holding contributing patients, glial cells, tumor sampling counts, and the interaction testing set fixed. Immune-like cells were excluded from both definitions. The earlier all-malignant sensitivity analysis is reported separately (Section 2.9; Supplementary Table S13A–C).

Marker panels used to refine astrocyte and myeloid identities within the TME are summarized in Table 1. These panels define sensitivity populations; the full scoring, anchor-gene, and competing-lineage criteria are described in Section 2.11.

Astrocyte calls require an anchor among *ALDH1L1*, *AQP4*, *SLC1A2*, and *SLC1A3*; the non-transporter sensitivity uses *ALDH1L1*/*AQP4*. The microglial anchor is *P2RY12*, *TMEM119*, *GPR34*, or *SALL1* together with broad myeloid support. Composite panels, matched-null enrichment, competing-lineage exclusions, neighborhood or human-reference support, and QC criteria increase specificity at the expense of sensitivity. Activation and nuclear dropout can remove true cells; unresolved labels preserve this uncertainty. Shared activation genes are not proof of ontogeny. The accompanying annotation archive provides competing OPC, oligodendrocyte, neuronal, vascular, lymphoid, and dendritic panels and marker rationale.

Panel selection was informed by the astroglial transcriptome studies of Cahoy et al. (2008) and Zhang et al. (2014), microglial identity work by Butovsky et al. (2014), glioma myeloid studies by Pombo Antunes et al. (2021) and Müller et al. (2017), and the human CNS-border atlas of Sankowski et al. (2024); see references [12,13,24,27,28,34]. The latter supplied the human reference signatures. These sources support marker rationale; the composite inclusion rules are operational criteria defined in Section 2.11. Marker overlap is not independent validation. Complete rules, donor-level identity counts, and frozen cell IDs are retained in the accompanying annotation archive. The GSE registry contains only the 516 source TME nuclei; malignant labels are unchanged. Overlapping sensitivity sets must not be concatenated as independent cells.

### 2.5. Supplementary Cell2Sentence Analyses

Each of the 516 TME nuclei was matched by cell identifier to a saved Cell2Sentence record containing up to 1000 rank-ordered genes and a predicted label. The archived run template specifies vandijklab/C2S-Scale-Gemma-2-27B, human input, seed 42, batch size 8, at most 4096 input tokens, 24 generated tokens, and greedy decoding. It uses 4-bit NF4 quantization with double quantization and bfloat16 computation. Its prompt asks for a cell type from genes ordered by decreasing expression. The template is retained verbatim, including input-path placeholders; the exact TME execution log, immutable model revision, and installed package versions were not preserved. These settings describe the supplied implementation and cannot establish exact reconstruction of the original inference environment (Supplementary Table S18).

For the comparison of cell-type annotations, Cell2Sentence predictions and published GEO annotations were mapped to the following shared broad-lineage categories: Astrocyte, OPC/oligodendroglial, Microglia/myeloid, Endothelial, Pericyte/vascular, Neuronal, Other immune, and Other/ambiguous. All 516 nuclei were included, with no exclusion based on prediction confidence. Overall concordance and per-category precision, recall, and F1 scores were calculated using the GEO categories as the reference for calculation.

For the rank-weighted marker analysis, gene positions within each top-1000 cell_sentence were used to calculate rank-weighted marker scores. A marker appearing at rank (r) among the top 1000 Cell2Sentence genes received weight ((1001−r)/1000), whereas absent markers received weight 0. The per-cell score was the mean weight across all genes in the marker set. The implementation is available in the archived analysis code. The Cell2Sentence framework was cited as the methodological basis for representing rank-ordered gene-expression profiles as textual sequences [35].

The ranked-gene comparison evaluates whether a different representation highlights related gene patterns; it does not provide evidence from an independent sample. Confidence thresholds were not applied to the 516 saved labels. Exact prompts, label harmonization, saved output identifiers, and code checksums are provided in Table S18; package and model-revision gaps are reported rather than reconstructed retrospectively.

### 2.6. Conventional Astrocyte State-Program Scoring

All 47 astrocytes from the 11 tumor samples containing at least one astrocyte were analyzed using the complete raw-count matrix. Four literature-curated, mutually nonoverlapping gene sets were defined before scoring: homeostatic, pan-reactive, inflammatory, and ECM-remodeling. The complete gene sets are reported in Table 2 and Supplementary Table S2.

Within each nucleus, genes were ranked in decreasing order of raw count, with average ranks assigned to ties and ranks capped at 1500. Program scores were calculated using an implementation equivalent to the normalized Mann–Whitney U statistic used by UCell [36,37]. Because UCell scores are continuous and no universal positivity threshold is prescribed, continuous scores were retained for cell-level analyses. For the cell-level astrocyte program-score heatmap, scores were standardized separately within each program across all 47 astrocytes.

For the descriptive overlap summaries, each cell–program combination was assigned a threshold-based positive or negative call. Each observed score was compared with a null distribution generated from 10,000 matched random signatures. For every program gene, a control gene was sampled from the 200 eligible genes with the most similar astrocyte detection frequency and mean capped rank. Random sampling used a fixed seed of 20260806. A program was called positive in each cell when its observed score exceeded the cell-specific 95th percentile of the matched-null distribution and at least two genes from that program were detected. Calls were made independently for the four programs, allowing an astrocyte to be positive for multiple programs or for none. The two-gene criterion was an analysis-specific safeguard against a call driven by a single detected transcript and is not a standard UCell requirement.

Sensitivity analyses varied the custom detection requirement to one or three genes while retaining the same 47 nuclei, gene sets, scores, and cell-specific null cutoffs; no null calibration was refitted (Supplementary Table S8).

These threshold-based calls were used descriptively and were not adjusted for multiple testing or externally validated as cell-state classifications. A negative call did not indicate biological inactivity, absence of program-gene expression, or loss of astrocyte identity. In the program-score heatmap, individual astrocytes were grouped visually by tumor sample, with white gaps separating samples.

### 2.7. Microglial Program Scoring

Astrocyte and microglial scoring addressed different descriptive questions. Rank-based astrocyte scoring reduced dependence on library size and supported within-cell matched-null comparisons in a small population. Gene-wise standardization in the larger source microglial population summarized relative program variation across nuclei and donors. All 288 source-labeled microglial nuclei were evaluated using nine prespecified programs: homeostatic, inflammatory-associated, immunoregulatory-associated, interferon-response, phagocytic/lipid-associated, antigen-presentation-associated, hypoxia/stress-associated, complement, and proliferation-associated expression. These are gene-set descriptions rather than validated functional states. Table 3 provides retained genes; the complete requested and excluded gene sets are in Supplementary Table S10. Rank-based and unfiltered-gene sensitivity analyses assess dependence on the primary scoring choice (Section 2.11).

Before microglial module scoring, genes detected in fewer than nine of the 288 source-labeled microglial nuclei were excluded (<3% detection). Excluded genes were *IL6* (six nuclei), *CXCL9* (four), *CXCL10* (five), *CXCL11* (three), and *NOS2* (one) from the inflammatory program; *ARG1* (eight), *CCL18* (four), and *MARCO* (eight) from the immunoregulatory program; and *CA9* (two) from the hypoxia/stress program. Table 3 lists the retained genes; all listed homeostatic and antigen-presentation genes were retained. The complete requested-versus-retained gene audit is provided in Supplementary Table S10. Retained genes were normalized as ln(1 + CP10K), standardized across all 288 nuclei using gene-wise sample standard deviations, and averaged without weighting within each module. These are cohort-relative expression scores, not measurements of biological activity.

Inflammatory-associated and immunoregulatory-associated scores were displayed as continuous variables in a cell-level scatterplot. Zero is the cohort mean on the gene-standardized scale and was not used to define positive cells or discrete polarization categories in the revised analysis.

For descriptive visualization, each nucleus was labeled with the program having the highest mean gene-level z-score. This highest-relative-score label is a visualization convention, not a dominant biological function; multiple programs may coexist. We assessed gene-set sensitivity by omitting each retained gene in turn while keeping the baseline standardization scale fixed (Supplementary Table S15).

The same nine program concepts were also evaluated using the top 1000 ranked genes from Cell2Sentence. Per-cell rank-weighted scores and pooled group means were calculated as a supplementary comparison and are reported in Supplementary Figure S2. The genes used for the primary normalized-expression analysis, their scoring eligibility, and the smaller representative marker set used for visualization are listed in Table 3.

### 2.8. Pseudobulk Aggregation and Astrocyte Support-Module Scoring

Raw counts were summed computationally within each tumor sample to generate separate astrocyte and state-resolved tumor pseudobulk profiles. Samples were eligible when they contained at least five nuclei classified as Astrocytes in the published GEO annotations and at least 20 tumor nuclei assigned to the eight malignant states defined in Section 2.4. Three samples met these criteria—ATRT-MYC-3, ATRT-SHH-4, and ATRT-TYR-2—comprising 29 astrocytes and 4036 tumor nuclei.

Eight candidate astrocyte support modules were defined before scoring: glutamate and glutamine handling, lactate and glucose metabolism, lipid and cholesterol handling, antioxidant and glutathione biology, cytokine and growth-factor secretion, extracellular matrix and cell contact, extracellular-vesicle biology, and mitochondrial-transfer-associated machinery. Module genes are listed in Supplementary Table S3. Within each pseudobulk, counts were converted to counts per million and transformed as log_2_(CPM + 1); each module score was the unweighted mean transformed expression of its constituent genes. Paired differences were calculated as the astrocyte score minus the corresponding tumor score from the same sample. Means used genes present in the count matrix. *GPX1* was unavailable, so the antioxidant/glutathione module used fourteen of fifteen requested genes. Table S12 reconciles the available-gene results with the alternative requested-panel denominator; the other seven modules are unchanged.

### 2.9. Ligand–Receptor Inference

Ligand–receptor associations were inferred using CellChat 2.1.2 in R 4.4.3 with its bundled human database, encompassing secreted signaling, cell–cell contact, and extracellular-matrix–receptor interactions [38,39]. Input expression was normalized to counts per 10,000 and transformed as ln(1 + CP10K). Communication scores were calculated using truncated-mean averaging (trim = 0.1), with population.size = FALSE, seed 42, and 10,000 permutations.

Cell populations and donor balancing. The primary tumor definition comprised the eight published transcriptionally resolved malignant states. Astrocytes and microglia were identified using the deposited labels, with conservative marker-defined populations evaluated separately. All source malignant assignments were preserved.

For pooled subtype analyses, donors contributed to the tumor and microglial groups independently if they provided at least 150 tumor or 10 microglial nuclei, respectively. Within each subtype and cell group, qualifying donor contributions were downsampled to the smallest qualifying contribution. Astrocyte groups required at least five nuclei and comprised SHH-4 (seven nuclei), TYR-2 (12), and MYC-3 (10). Astrocytes were not downsampled because only one donor qualified per subtype. Accordingly, astrocyte donor effects could not be distinguished from subtype effects, and tumor and microglial donor contributions could differ.

Sensitivity to tumor inclusion. To assess the effect of including Unannotated malignant nuclei, two tumor definitions were compared separately within MYC, SHH, and TYR: the eight resolved malignant states and those states plus Unannotated nuclei. Immune-like malignant cells were excluded from both definitions. The comparisons retained identical contributing donors, astrocyte and microglial cells, and per-donor tumor sampling targets of 184 nuclei for MYC, 870 for SHH, and 292 for TYR. Tumor nuclei were sampled without replacement from each eligible donor pool. All six models tested the same 3220 database interactions with complete ligand and receptor gene components represented in the expression matrix, independently of overexpression screening. BH correction covered 28,980 source–target–interaction entries per model. This design evaluates tumor inclusion under one reproducible balanced sampling realization (Table S13A,C).

A separate analysis compared the resolved-state definition with all published malignant populations, including Unannotated and Immune-like cells, using the same eligibility thresholds and balancing procedure. Donor eligibility and sampling counts could therefore change. These models used interaction families selected by CellChat’s overexpression screening and assessed sensitivity to the broader tumor definition (Table S13B).

Donor-level and annotation sensitivity analyses. Individual-donor models required at least 20 state-resolved tumor nuclei and, for the relevant interaction, at least 10 microglial nuclei or five astrocytes. Additional models evaluated conservative cell identities and inclusion of MYC-2. In the latter analysis, MYC-2 contributed 18 eligible microglial nuclei, whereas its tumor and astrocyte populations remained below the inclusion thresholds. Minimum TME group sizes of five, 10, and 20 were also examined by post-fit eligibility filtering, retaining the tumor minimum of 20 and the microglial minimum of 10. This filtering assessed population support without repeating interaction selection or changing the multiple-testing family. The five-astrocyte minimum was a permissive exploratory threshold rather than an established reliability criterion (Table S16).

Permutation testing and multiple-testing correction. All models used 10,000 permutations. Cell-group labels were temporarily shuffled within each model while preserving expression profiles and group sizes; stored annotations and donor identifiers were unchanged. Permutations in pooled models were not blocked by donor. Finite-permutation *p* values were calculated as (b + 1)/(10,000 + 1), where b is the number of permuted scores strictly exceeding the observed score; zero observed scores were assigned *p* = 1. Benjamini–Hochberg correction included every tested source–target–interaction entry within each model. Nominal support was defined as finite *p* < 0.05 and multiplicity-controlled support as BH q < 0.05. These tests quantify within-model support and do not establish differences between molecular subtypes or consistency across independent patients.

Receptor-complex and expression checks. Candidate interactions were mapped to exact CellChat identifiers and complete database receptor definitions, including *SPP1–(ITGAV + ITGB1)*. The literature-based candidate panel is provided in Table S4. Same-donor expression checks assessed ligand detection in source cells and simultaneous detection of all required receptor subunits in target nuclei. These analyses identify expression-supported interaction candidates rather than demonstrating functional signaling.

### 2.10. Statistical Analysis

Results were summarized using counts, percentages, means, medians, ranges, or paired differences, as appropriate. Recovered cell-type proportions and astrocyte program-positive percentages were summarized descriptively. Astrocyte program positivity was determined using the matched-null thresholds described in Section 2.6; these threshold-based calls were not adjusted for multiple testing. For visualization, astrocyte program scores were standardized separately within each program across all 47 nuclei. Matched astrocyte–tumor pseudobulk comparisons were summarized using within-sample differences and the median difference across the three eligible tumor samples. Given the limited number of matched tumors, these comparisons were treated as descriptive, and no inferential test was applied.

The cell-level Spearman correlation between inflammatory-associated and immunoregulatory-associated scores was descriptive, because nuclei from the same tumor are not independent patients. For molecular-subtype comparisons, source microglial scores were averaged within each tumor and compared with Kruskal–Wallis tests, followed by BH correction across nine programs. Tumor was the inferential unit. The small subtype groups (MYC, *n* = 3; SHH, *n* = 5; TYR, *n* = 4) limit power; non-significance does not demonstrate equivalence.

For the ATRT-MYC-2 sensitivity analysis, donor-level module means and Kruskal–Wallis/BH results were recomputed with and without this sample while retaining the original 288-nucleus gene-standardization scale. Composition summaries were also recomputed separately. *CD74* associations used donor-mean ln(1 + CP10K) expression and donor-mean module scores, with *CD74* removed from the antigen-presentation module before correlation. Two-sided Spearman permutation tests used 99,999 donor-label permutations (seed 20261007), with BH correction across nine modules within each sample scope. The analysis was repeated without ATRT-MYC-2. Cell-level correlations and rank-residual correlations adjusted for donor indicators and library depth were descriptive only (Supplementary Tables S9 and S11).

### 2.11. TME-Restricted Annotation and Scoring Sensitivities

Annotation sensitivities were designed to assess the dependence of downstream findings on population definitions, rather than to establish a single definitive annotation (Section 4.4). Only the 516 GSE TME nuclei entered annotation refinement. Raw-count UCell-like rank scores used a maximum rank of 1500 and average ranks for ties. Composite panels were calibrated against 2000 random signatures matched on detection and mean expression, drawing each control gene from its 200 closest eligible matches without duplicate genes within a signature (seed 20261005). Empirical *p* < 0.05, at least two detected panel genes, an appropriate anchor, and neighborhood or reference-signature support defined operational conservative calls. A competing lineage exceeding the focal enrichment z-score by more than one unit vetoed a call. At least 200 detected genes, positive library size, and ≤10% mitochondrial counts were required. These analysis-specific cutoffs favor specificity; their empirical *p* values are not cell-identity error rates. Marker panels and anchor requirements are summarized in Table 1; complete rules and exact cell IDs are retained in the accompanying annotation archive.

Astrocyte markers included *ALDH1L1*, *AQP4*, *SLC1A2*, *SLC1A3*, *GFAP*, and *SOX9*, with an anchor among the first four. Microglial markers included *P2RY12*, *TMEM119*, *GPR34*, *SALL1*, *P2RY13*, *SLC2A5*, *OLFML3*, and *CX3CR1*, with an anchor among the first four and at least two broad myeloid backbone genes. Macrophage-associated, monocyte-associated, and border-associated macrophage (BAM)-like panels were considered separately. Positive competing myeloid evidence prevented assignment to a pure microglial population; ambiguous profiles remained unresolved. Panels combine identity-associated genes with competing-lineage checks because *GFAP*, *SLC1A3*, *CD163*, or *SPP1* alone is insufficient. Literature and human reference signatures supported panel selection [11,12,13,24,27,28,30,34]. Reference-signature enrichment, using the published human CNS atlas tables, is an overlapping marker check and not independent label-transfer validation. Macrophage-like and BAM-like names do not establish blood-derived origin or anatomical location.

Conservative astrocytes were the intersection of source astrocytes and marker-supported cells; an expanded set admitted additional supported nuclei only from the source TME. Sensitivities varied identity detection requirements to one or three genes and used a non-transporter panel (*ALDH1L1*, *AQP4*, *GFAP*, *SOX9*, *S100B*, *FGFR3*) with *ALDH1L1*/*AQP4* anchoring. The latter reduces circular selection by *SLC1A2*/*SLC1A3* when studying glutamate-associated expression. Astrocyte programs and paired pseudobulk modules were reevaluated within each set. Microglial comparisons used fixed baseline z-scores, rank scores, and scores without the nine-cell gene filter; subtype tests required at least two eligible donors per subtype, each with at least five relevant nuclei. Single-gene omission assessed sensitivity of the highest-relative-score display. These analyses assess robustness, not annotation accuracy (Supplementary Figures S4 and S5; Table 1 and Table S15).

### 2.12. External ScPCA Cohort

Eight ATRT nuclear libraries from ScPCA project SCPCP000010 represented paired initial and progressive or recurrent specimens from four patients [40]. The downloaded package was generated on 29 September 2026 and was processed by scpca-nf v0.9.3 (commit 80e7bbb78202ba6cb618feab59af433c750fcdc7), using GRCh38.104 and 10x v2 5-prime data. We analyzed total-RNA integer counts from the raw layer, merged duplicate gene symbols by summation, and retained library, specimen, and patient identifiers. Supplied disease timing defines patient_group; it is not molecular subtype. MYC/SHH/TYR labels were not available. Clinical metadata are reported as provided, including whether age refers to diagnosis or collection (Supplementary Table S14).

The frozen external annotation used the same composite-marker principles with cohort-specific calibration and compartment safeguards. Predicted doublets, nuclei with fewer than 200 genes or >10% mitochondrial counts, and empty libraries were excluded. Named TME populations required coherent markers and normal-compatible source inferCNV evidence, or marker-supported immune/vascular reference cells where CNV was unavailable. Astrocytes additionally required a non-transporter panel, *ALDH1L1*/*AQP4* detection, and no coherent myeloid conflict. Source SingleR, CellAssign, and SCimilarity predictions were supporting evidence, not a vote establishing malignancy. Conservative tumor candidates exceeded the sample normal-reference 95th-percentile thresholds for CNV count and mean affected-arm fraction, had at least two arms with ≥50% affected genes, ≥80% high-CNV neighbors, a source cluster of ≥20 QC-passing nuclei, and no strong immune/vascular conflict. The broader sensitivity relaxed CNV thresholds to the 90th percentile and neighbor support to 50%, while retaining conservative members. A library without source inferCNV was excluded from tumor analyses; expression-only tumor candidates were not promoted to the primary group. Exact thresholds and cell IDs are in Table S17.

External CellChat models were fitted separately for each specimen with a CNV-supported tumor group and eligible TME groups. Primary conservative models required ≥20 nuclei per group; sensitivities used broader annotation at 20 and conservative annotation at 10. Minimum counts were inclusive; because CellChat removes groups with counts ≤ its internal cutoff, the internal cutoff was set one below the stated minimum (implementation audit in Table S17). Full-library totals normalized raw expression as ln(1 + CP10K). Settings were CellChat 2.1.2 with its full human database, truncatedMean, trim 0.1, population.size = FALSE, 10,000 permutations, and seed 20261008. We reported native *p*, finite-permutation *p* = (b + 1)/10,001, and BH adjustment over all source–target–interaction entries in each model. *APP–CD74*, *SPP1–CD44*, *SPP1–(ITGAV + ITGB1)*, *NCAM1–FGFR1*, and *PTN–PTPRZ1* were specified before these fits. Candidate recurrence was summarized by specimen and distinct patient, distinguishing ineligible, unselected, tested non-nominal, and BH-supported results. Model-level *p* values do not test patient-level recurrence.

External expression analyses reused the retained discovery microglial gene sets with *CD74* excluded from correlated modules. Gene-wise z-scores were recalibrated within the external operational myeloid compartment; cross-cohort score magnitudes were not compared. Groups with ≥20 nuclei were summarized by specimen, then eligible specimen means were equally averaged within each patient. *CD74* correlations used exact two-sided permutations of patient labels and BH correction across nine programs; groups with fewer than three patients were not tested. The pooled operational myeloid analysis includes the explicitly unresolved group and is not a microglia-specific validation.

### 2.13. Use of Generative AI

The authors used OpenAI Codex GPT-5.6 and GPT-6 (OpenAI, San Francisco, CA, USA; accessed February and October 2026) to assist with computational analysis based on instructions, scientific content, and interpretations provided by the authors. R coding was assisted by Anthropic Claude Sonnet 4.6 (Anthropic, San Francisco, CA, USA), which helped streamline analysis pipeline development and troubleshooting throughout the study. The revised analysis package provides code, input provenance, cell identifiers, and model outputs for author review and reproducibility. The authors take full responsibility for the accuracy, originality, validity, and integrity of the published work.

## 3. Results

### 3.1. Cellular Landscape of the ATRT Tumor Microenvironment

**Figure 2:**
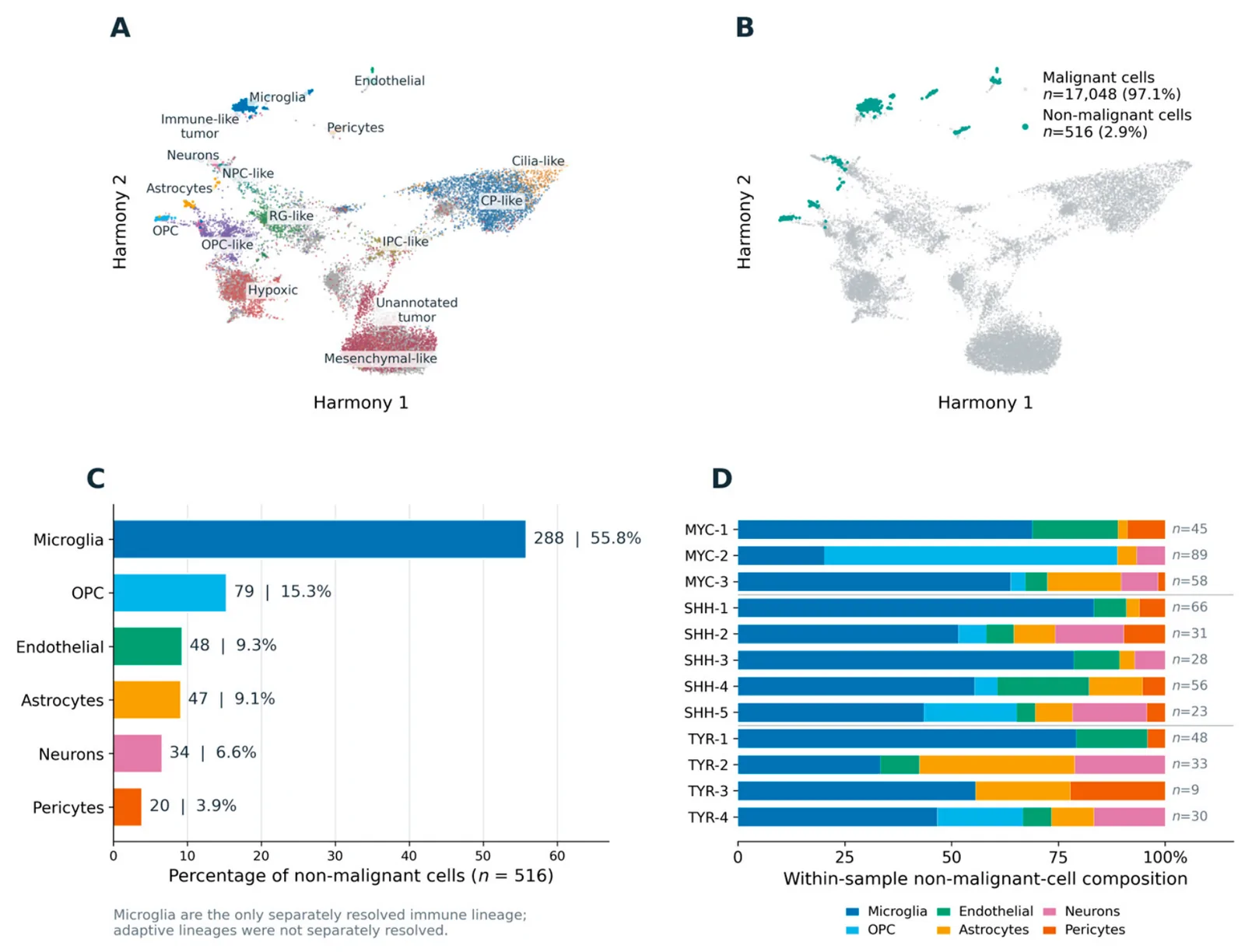
Recovered malignant and nonmalignant populations in GSE283839. (**A**) Deposited Harmony embedding of 17,564 nuclei with source labels. (**B**) The 516 source TME nuclei are highlighted; 17,048 author-classified malignant nuclei are gray. No additional filtering or embedding was applied. (**C**) Source TME labels comprise 288 Microglia (55.8%), 79 OPC (15.3%), 48 endothelial (9.3%), 47 astrocyte (9.1%), 34 neuronal (6.6%), and 20 pericyte nuclei (3.9%). “Microglia” is the original label, not proof of resident origin. (**D**) Within-sample recovered TME composition; labels give TME counts. The verified range is 9–89 nuclei per tumor, so small denominators can produce unstable proportions. These results describe recovered nuclei, not intact-tissue abundance. Conservative identity counts are reported in Section 3.6 and the accompanying annotation cell registry; marker criteria are summarized in Table 1.

In total, 17,564 nuclei from 12 ATRT tumors spanning the ATRT-MYC, ATRT-SHH, and ATRT-TYR molecular subtypes were analyzed. Cell annotations and Harmony coordinates were obtained from the published single-nucleus transcriptomic atlas of Blanco-Carmona, Paassen, He, et al. [2] and used to visualize the cellular landscape of the cohort (Figure 2A). Based on the published annotations, 17,048 nuclei (97.06%) were assigned to the tumor compartment and 516 (2.94%) to the nonmalignant tumor microenvironment (TME) (Figure 2B,C; Supplementary Table S1). The tumor compartment included 7676 Unannotated nuclei, which were classified in the original study as tumor nuclei without a specific tumor-state signature.

The source Microglia label was the largest recovered nonmalignant group (288/516; 55.81% of TME and 1.64% of all nuclei). Other deposited TME labels included OPC (79), endothelial cells (48), astrocytes (47), neurons (34), and pericytes (20) (Figure 2C). The deposited framework did not separately resolve adaptive lymphocytes or natural killer cells; their absence as named categories cannot establish biological absence. Marker refinement subsequently identified substantial uncertainty within the source microglial category (Section 3.6).

Recovered nonmalignant fractions varied markedly by sample (median 3.33%; range 1.32–87.25%). ATRT-MYC-2 exhibited the highest proportion of nonmalignant cells, with 89 of 102 recovered nuclei classified as nonmalignant.

These small and uneven counts preclude interpreting the pooled source microglial predominance as a general property of ATRT tissue. The data also cannot classify ATRT as immunologically cold, because immune context requires information on lymphocyte density, spatial organization, and activity.

The recovered TME count ranged from nine nuclei in ATRT-TYR-3 to 89 in ATRT-MYC-2. Removing ATRT-MYC-2 reduced the pooled MYC TME proportion from 192/3806 (5.04%) to 103/3704 (2.78%). Thus, this sample materially affects composition estimates, even though its low tumor recovery prevents its inclusion as a tumor contributor under the CellChat thresholds (Supplementary Table S9).

### 3.2. Astrocytes Show Overlapping, Sample-Variable State Programs

**Figure 3:**
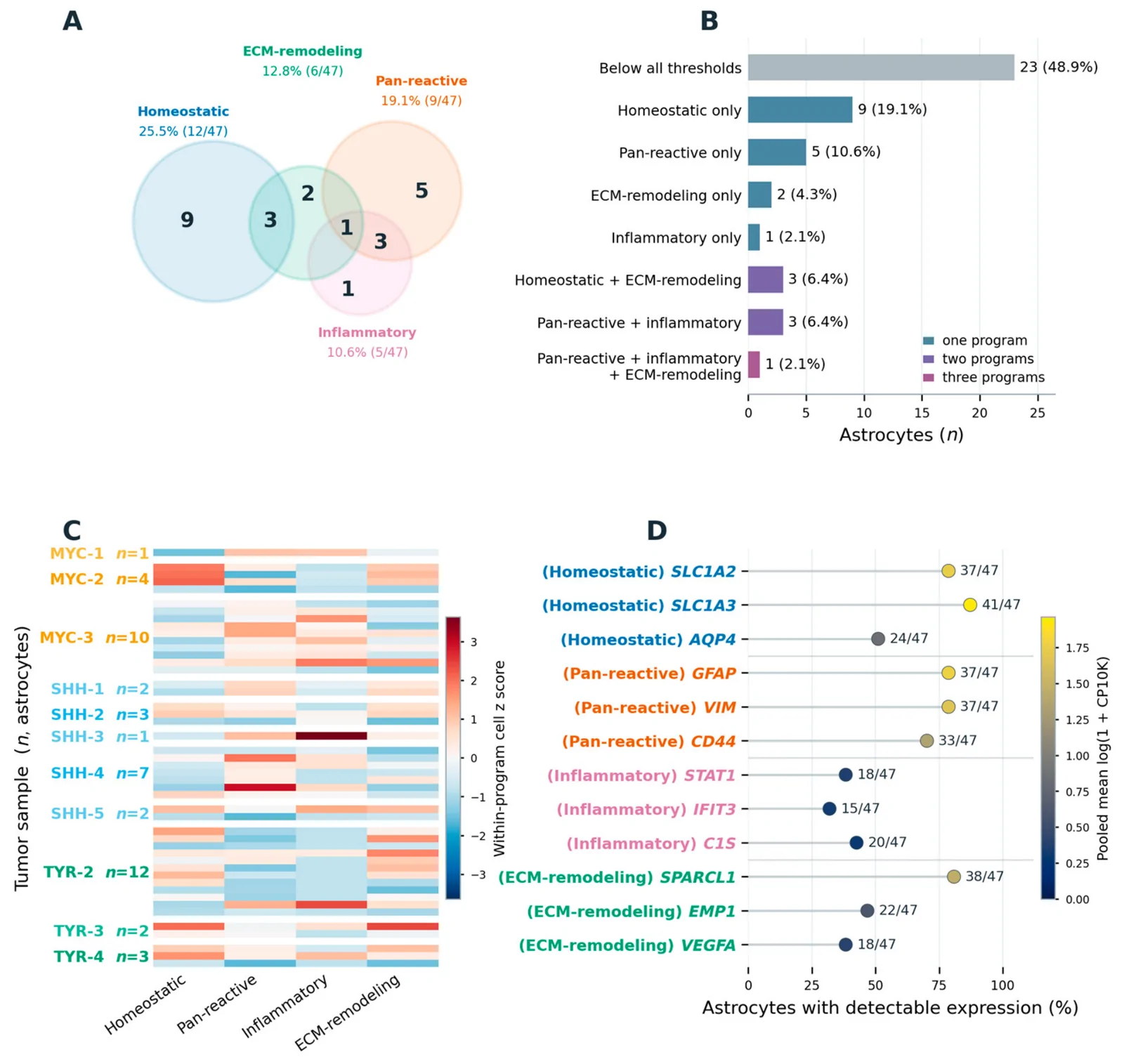
Heterogeneous astrocyte transcriptional programs across ATRT tumors revealed by conventional full-matrix analysis. The four literature-curated astrocyte programs—homeostatic, pan-reactive, inflammatory, and ECM-remodeling—were evaluated across 47 GEO-annotated astrocyte nuclei from 11 ATRT tumor samples. (**A**) Area-proportional summary of nominal program-positive astrocytes pooled across all tumors. Numbers within overlap regions indicate exact cell counts; overlap geometry is illustrative and not quantitative. (**B**) Distribution of astrocytes according to nominal program membership. Cells below all thresholds represent cells not meeting any predefined criterion and do not indicate absence of astrocyte identity or biological activity. (**C**) Heatmap of program scores for individual astrocytes. Each row represents one nucleus, grouped by tumor sample, and colors indicate within-program standardized scores across the 47 cells. Samples with limited astrocyte recovery are shown for completeness but are not interpreted as independent patient-level estimates. (**D**) Detection prevalence and pooled expression levels of representative genes contributing to each program. Dot position indicates the percentage of astrocytes with detectable expression, labels indicate positive-cell counts, and dot color represents pooled mean log(1 + CP10K). These programs represent overlapping transcriptional states and do not define discrete astrocyte identities or functional phenotypes. Requiring at least one or three detected signature genes instead of two, with the null thresholds held fixed, leaves all program-positive calls and overlap categories unchanged (Supplementary Table S8).

Across 47 GEO-annotated astrocytes from 11 tumor samples, conventional full-matrix rank scoring identified evidence for multiple astrocyte state programs. A cell was considered positive for a program when its score exceeded the 95th percentile of 10,000 expression-matched random gene signatures and at least two genes from that program were detected. Using this threshold, 12 astrocytes (25.5%) were positive for the homeostatic program, nine (19.1%) for pan-reactive, five (10.6%) for inflammatory, and six (12.8%) for the ECM-remodeling program (Figure 3A).

The four programs showed substantial overlap (Figure 3B). Among the 47 astrocytes, 23 cells (48.9%) did not meet the threshold for any program, 17 (36.2%) were positive for one program, six (12.8%) for two programs, and one (2.1%) for three programs. Specifically, nine cells were positive only for the homeostatic program, five only for pan-reactive, one only for inflammatory, and two only for the ECM-remodeling program. Three cells were positive for both homeostatic and ECM-remodeling programs, three for both pan-reactive and inflammatory programs, and one for pan-reactive, inflammatory, and ECM-remodeling programs. Cells below all four thresholds may still express genes associated with these programs; therefore, this category does not indicate biological inactivity or loss of astrocyte identity.

The cell-level standardized heatmap included all 47 astrocytes and grouped cells according to tumor sample, revealing substantial heterogeneity both within and across tumors (Figure 3C). Consistent with this heterogeneity, individual program-associated genes were detected at different frequencies across the pooled astrocyte population. *SLC1A3* was detected in 41 of 47 astrocytes, *SLC1A2* and *GFAP* in 37 of 47, *VIM* in 37 of 47, and *SPARCL1* in 38 of 47, whereas inflammatory-associated markers were detected in smaller subsets (Figure 3D).

The custom detection safeguard was evaluated with the original 10,000 matched-null calibrations and 95th-percentile cutoffs held fixed. Requiring at least one, two, or three detected signature genes produced identical calls for every astrocyte and every program: homeostatic 12/47 (25.5%), pan-reactive 9/47 (19.1%), inflammatory 5/47 (10.6%), and ECM-remodeling 6/47 (12.8%). Program overlaps and the 23/47 nuclei below all program thresholds were also unchanged (Supplementary Figure S3A; Supplementary Table S8). This establishes stability to this particular detection threshold, not independent validation of the annotations or biological states.

As a supplementary comparison of the same data, Cell2Sentence and GEO labels agreed for 376/516 TME nuclei (72.9%) after broad-lineage harmonization (Supplementary Figure S1; Table S6). Among 47 source astrocytes, 30 were predicted astrocyte-like by Cell2Sentence: 21/24 above at least one astrocyte program threshold and 9/23 below all thresholds. The remaining below-threshold nuclei included 12 OPC/oligodendroglial predictions, one neuronal prediction, and one ambiguous prediction. This disagreement motivated the independent-of-Cell2Sentence marker workflow; neither classifier was treated as ground truth. The marker audit uses overlapping expression evidence and remains an annotation sensitivity analysis (Section 3.6).

### 3.3. Donor-Specific Astrocyte Expression and Ligand–Receptor Candidates

**Figure 4:**
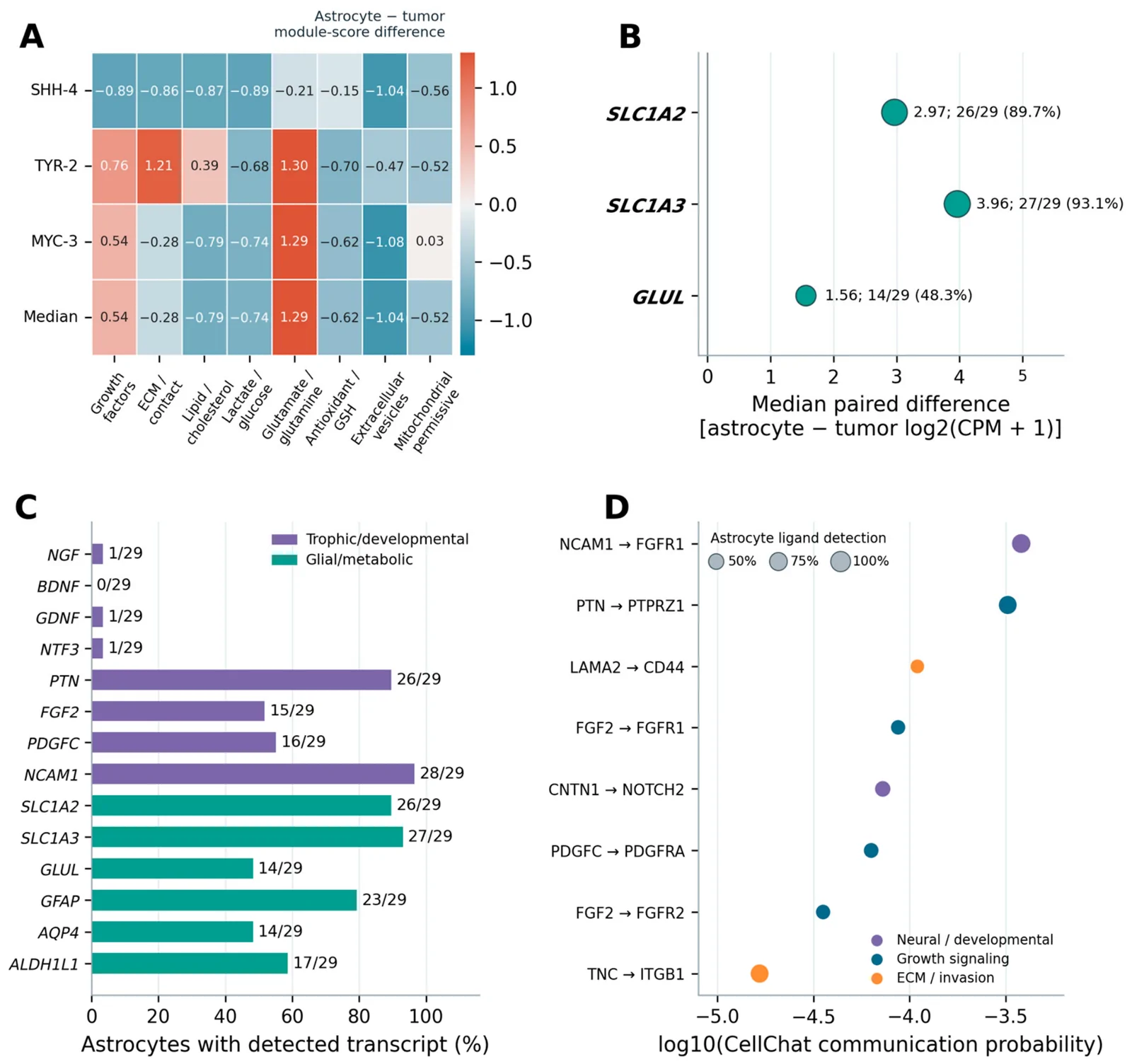
Astrocyte expression modules and candidate astrocyte–tumor associations. (**A**) Astrocyte-minus-matched-tumor module-score differences for ATRT-SHH-4, ATRT-TYR-2, and ATRT-MYC-3, with the across-sample median shown. Positive values indicate higher astrocyte module scores; comparisons include three tumors and are interpreted descriptively. (**B**) Median paired astrocyte-minus-tumor expression differences for *SLC1A2*, *SLC1A3*, and *GLUL*. Dot size and adjacent labels indicate transcript detection among the 29 astrocytes. (**C**) Transcript detection frequencies among the same 29 astrocytes. Classical neurotrophins were sparsely detected, whereas developmental, matrix-associated, and glial-state-associated transcripts showed broader detection. (**D**) Selected pooled astrocyte-to-tumor CellChat candidate interactions. Horizontal position represents log10 communication probability, dot size indicates astrocyte ligand-detection frequency, and color denotes the prespecified biological theme. The initial displayed values and literature-based context are provided in Supplementary Table S5. The glutamate/glutamine module differences were +1.303 (TYR-2), +1.294 (MYC-3), and −0.211 (SHH-4), emphasizing donor variation. All functional labels denote expression-associated hypotheses, not measured metabolic flux or active signaling. Panel D retains the initial pooled display; receptor shorthand is not evidence of a complete receptor complex. Analyses using complete receptor definitions and same-donor expression checks are described in Section 3.5, Supplementary Figure S6, and Tables S13 and S16.

Matched pseudobulk analysis was possible for only three tumors containing at least five astrocytes and 20 state-resolved tumor nuclei (29 astrocytes and 4036 tumor nuclei overall). The median astrocyte-minus-tumor glutamate/glutamine-module difference was +1.294 log2(CPM + 1), but the individual differences were +1.303 in ATRT-TYR-2, +1.294 in ATRT-MYC-3, and −0.211 in ATRT-SHH-4 (Figure 4A; Supplementary Table S12). Thus, the positive median reflects two tumors and does not indicate a uniform feature of ATRT-associated astrocytes. The cytokine/growth-factor module had a median difference of +0.544, while ECM/contact had a median difference of −0.277; the remaining support modules were lower in astrocytes at the median. These three-donor comparisons are descriptive and hypothesis-generating.

At the individual gene level, coordinated expression of *SLC1A2*, *SLC1A3*, and *GLUL* provided the strongest transcriptomic support for a glutamate–glutamine metabolism hypothesis (Figure 4B). *SLC1A3* was detected in 27/29 astrocytes (93.1%) and showed a median astrocyte-minus-malignant-cell difference of +3.96 log2(CPM + 1). *SLC1A2* was detected in 26/29 astrocytes (89.7%; median difference +2.97), and *GLUL* in 14/29 astrocytes (48.3%; median difference +1.56). Figure 4B displays these median paired differences, while Figure 4A preserves sample-level variation across the three tumors. These expression patterns motivate hypotheses about glutamate handling and glutamine synthesis; they do not establish enhanced uptake, synthesis, metabolic flux, or nutrient transfer to tumor cells.

A focused analysis of classical neurotrophic factors did not identify a dominant neurotrophic expression program in ATRT-associated astrocytes (Figure 4C). *NGF*, *GDNF*, and *NTF3* were each detected in only 1/29 astrocytes (3.4%), and *BDNF* was not detected. In contrast, several neural-developmental and growth-associated transcripts were broadly detected, including *NCAM1* (28/29, 96.6%), *PTN* (26/29, 89.7%), *PDGFC* (16/29, 55.2%), and *FGF2* (15/29, 51.7%). Canonical glial support transcripts remained detectable, including *SLC1A3* (93.1%), *SLC1A2* (89.7%), *GFAP* (79.3%), *ALDH1L1* (58.6%), *GLUL* (48.3%), and *AQP4* (48.3%).

In the initial population-size-weighted pooled CellChat analysis, *NCAM1–FGFR1* and *PTN–PTPRZ1* showed the highest displayed astrocyte-to-malignant-cell communication probabilities (log10 probabilities −3.42 and −3.49, respectively; Figure 4D; Supplementary Table S5). Additional retained candidate interactions included *LAMA2–CD44*, *FGF2–FGFR1/2*, *CNTN1–NOTCH2*, *PDGFC–PDGFRA*, and *TNC–ITGB1*. *NCAM1–FGFR1* was retained across the three initial subtype objects. Each object contained astrocytes from one donor, and this pooled observation alone does not establish that the ligand and receptor are expressed within the same tumor or that the candidate is subtype-specific. These legacy display labels are retained for provenance; integrin-chain shorthand does not establish complete receptor identity.

The prespecified organelle-transfer-associated expression module was not enriched. *GAP43*, *GJA1*, and *RHOT1* have broader cellular roles, so their detection provides no evidence of mitochondrial transfer in ATRT.

### 3.4. Overlapping Source Microglial Transcriptional Programs

**Figure 5:**
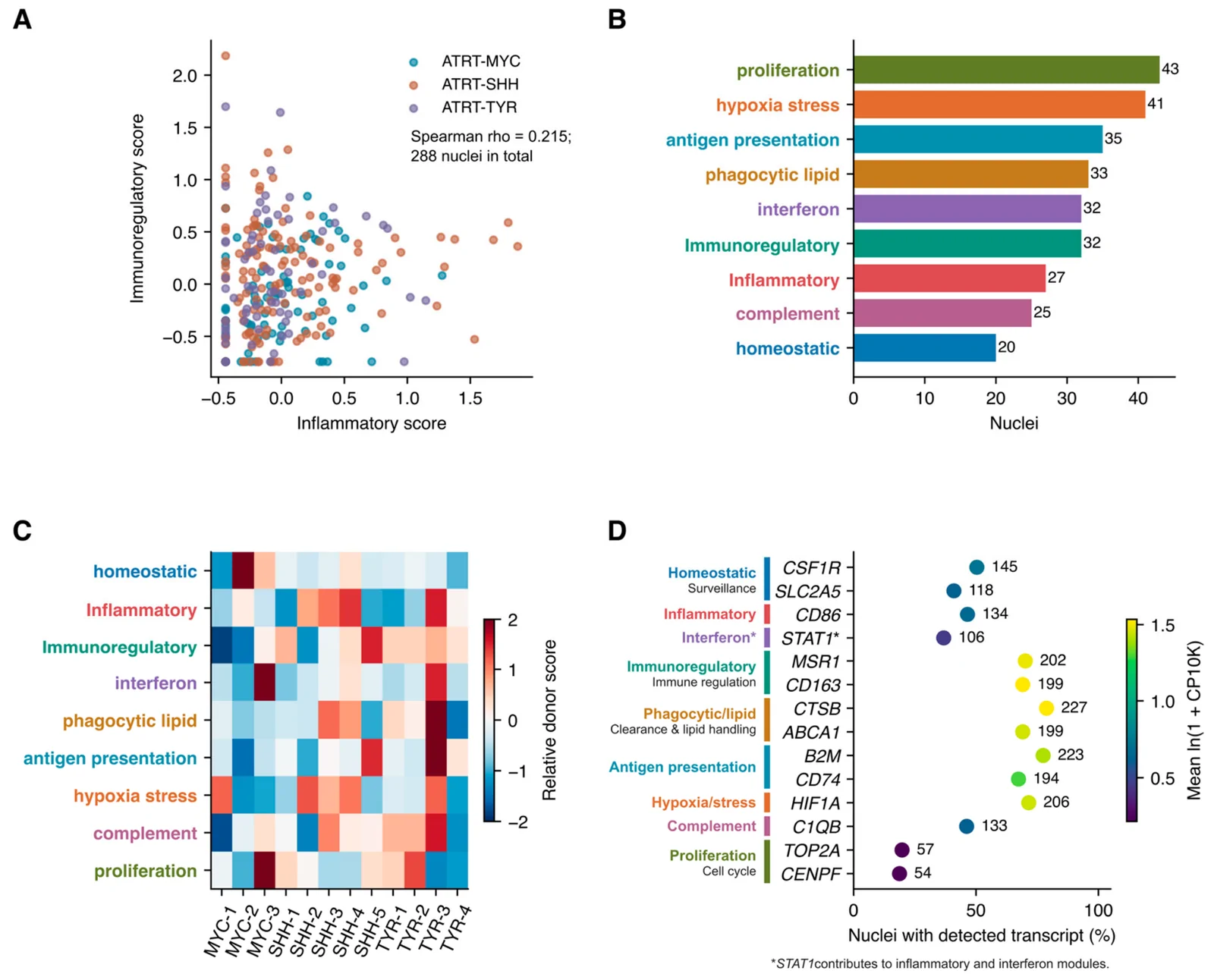
Relative expression programs in 288 source-labeled microglial nuclei. (**A**) Continuous inflammatory-associated and immunoregulatory-associated scores, colored by subtype. The correlation is descriptive; zero is not a biological cutoff. (**B**) Highest-relative-score assignments are for display only; programs may coexist and do not establish dominant functions. (**C**) Donor means standardized within each program. Each column represents one tumor (nucleus counts: Supplementary Table S9). Subtype differences were not statistically detectable (BH-adjusted *p* ≥ 0.663 across nine programs). (**D**) Representative transcript detection percentages, positive-cell counts, and mean ln(1 + CP10K) expression. Module colors match across (**B**–**D**); heatmap/dot colors indicate scores/expression. Expression does not establish function. Scoring and single-gene-omission sensitivities: Supplementary Table S15.

Inflammatory-associated and immunoregulatory-associated scores varied continuously across 288 source-labeled microglial nuclei and showed a weak positive cell-level correlation (Spearman rho = 0.215; Figure 5A). This descriptive association is compatible with overlapping expression programs. We did not classify cells by whether either centered score exceeded zero or infer discrete functional polarization states.

Marker-level detection further demonstrated heterogeneous source microglial expression (Figure 5D). Markers of the homeostatic program included *CSF1R* (50.3%) and *SLC2A5* (41.0%). Markers of the inflammatory-associated program included *CD86* (46.5%) and *STAT1* (36.8%), whereas markers of the immunoregulatory-associated program included *MSR1* (70.1%) and *CD163* (69.1%). Additional frequently detected markers included *CTSB* (78.8%) and *ABCA1* (69.1%) from the phagocytic and lipid-processing program; *B2M* (77.4%) and *CD74* (67.4%) from the antigen-presentation program; *HIF1A* (71.5%) from the hypoxia and stress-response program; and *C1QB* (46.2%) from the complement program. Proliferation-associated transcripts *TOP2A* and *CENPF* were detected in smaller subsets of the source microglial population (19.8% and 18.8%, respectively).

For the Figure 5B summary, the single highest relative module score was proliferative in 43 nuclei (14.9%), hypoxia/stress in 41 (14.2%), antigen presentation in 35 (12.2%), phagocytic/lipid-associated in 33 (11.5%), interferon response and immunoregulatory-associated in 32 each (11.1%), inflammatory-associated in 27 (9.4%), complement in 25 (8.7%), and homeostatic in 20 (6.9%). These mutually exclusive display labels summarize a continuous score matrix; they do not make the underlying programs mutually exclusive or identify a measured dominant cellular function.

A sample-by-module heatmap revealed substantial heterogeneity within and across ATRT samples (Figure 5C). No microglial module score showed a significant difference among ATRT-MYC, ATRT-SHH, and ATRT-TYR samples after Benjamini–Hochberg correction (all FDR ≥ 0.66), indicating that subtype-level differences were not detectable in this dataset.

Repeating the donor-level subtype analysis without ATRT-MYC-2 did not reveal a detectable program difference: BH-adjusted *p* values were at least 0.663 with all 12 tumors and at least 0.969 with 11 tumors. The sample was retained in the primary score calibration and Figure 5 summaries. This sensitivity result does not establish equivalence of the molecular subtypes or exclude meaningful biological differences (Supplementary Table S9).

### 3.5. Candidate Microglia–Tumor Ligand–Receptor Associations

**Figure 6:**
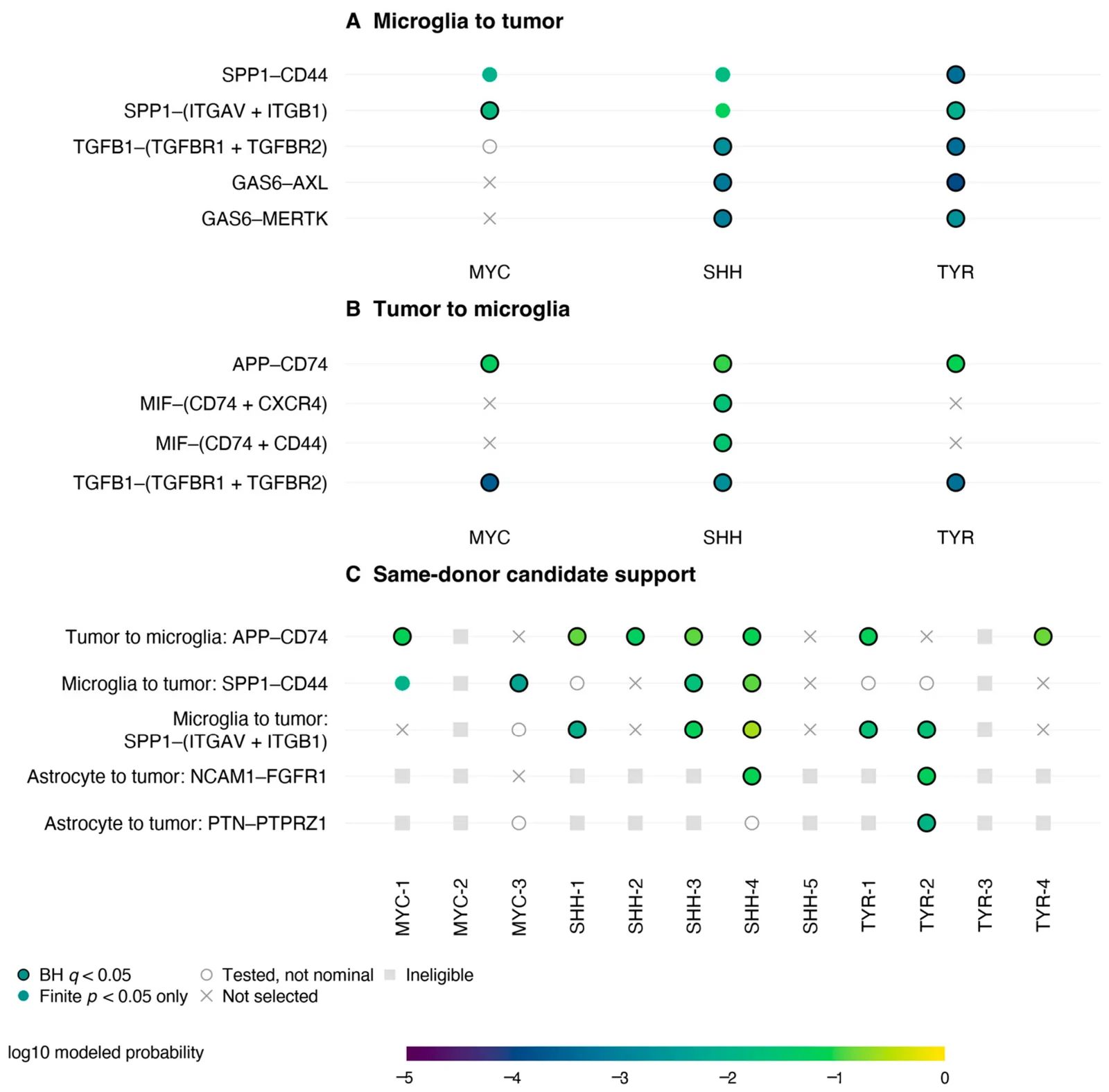
Fresh CellChat candidate associations with exact receptor definitions. (**A**,**B**) Source microglia-to-tumor and tumor-to-microglia predictions in state-resolved subtype models. (**C**) Five highlighted candidates in separate donor fits. All panels use 10,000 permutations. Colored circles denote finite-permutation *p* < 0.05; a black outline denotes within-model BH q < 0.05 in every panel. Gray open circles denote tested non-nominal candidates, crosses denote candidates not selected by the model, and gray squares denote ineligible groups. Unselected and ineligible candidates are not evidence of biological absence. Color indicates log10 modeled probability. Donor fits require ≥20 state-resolved tumor, ≥10 source microglial, and ≥5 astrocyte nuclei for the relevant groups. All models use CellChat 2.1.2, its bundled human database, truncatedMean with trim = 0.1, seed 42, and population.size = FALSE. BH adjustment covers every tested source–target–interaction entry in each model. Subtype contrasts remain qualitative; each astrocyte-qualified subtype has one contributing astrocyte donor. Tables S13 and S16 provide cell IDs, donor contributions, expression checks, and sensitivity results.

Fresh CellChat 2.1.2 fits nominated *SPP1*-related microglia-to-tumor and *APP–CD74* tumor-to-microglia candidates (Figure 6A,B). Exact receptor complexes replace the original single-chain shorthand. Probabilities vary with group definition and contributing donors; differences between subtype objects were not evaluated using a donor-level test of subtype effects. Finite-permutation nominal retention and within-object BH support are distinguished in Figure 6A,B after 10,000 permutations; all finite-permutation and adjusted values are provided in Table S13. 

In the fresh state-resolved subtype models, *APP–CD74* was retained in each of the three subtype objects (Figure 6B), while *MIF/CD74*-associated and *TGFB*-associated candidates varied between objects. This repeated computational prediction does not establish receptor activation or a conserved biological effect. To contextualize *CD74*, we correlated its donor-mean normalized expression with the nine microglial module scores, removing *CD74* from the antigen-presentation score. Across 12 tumors, the antigen-presentation association was strong (Spearman rho = 0.923; two-sided permutation *p* = 0.00005; BH-adjusted *p* = 0.00045), whereas the immunoregulatory association was weaker and did not pass correction (rho = 0.671; *p* = 0.02048; adjusted *p* = 0.09216). Excluding ATRT-MYC-2 retained the antigen-presentation association (rho = 0.909). Descriptive adjustment for donor and library depth at the nucleus level also retained a positive antigen-presentation association (partial rank correlation = 0.676). These findings link *CD74* expression to an antigen-presentation-associated transcriptional context, not to an experimentally established response to APP (Supplementary Figure S3B,C; Supplementary Table S11).

Same-donor expression checks supported the presence of candidate transcripts while revealing uneven detection. Across the 12 source samples, *APP* was detected in 40.0–96.9% of state-resolved tumor nuclei and *CD74* in 50.0–90.0% of source-labeled microglial nuclei. These descriptive ranges include small groups; two samples were ineligible for donor CellChat fitting. Supplementary Figure S6 shows donor-specific *APP*/*CD74*, *NCAM1*/*FGFR1*, and *PTN*/*PTPRZ1* expression. Supplementary Table S16 also reports complete-receptor co-detection, including multimeric integrins, rather than treating an individual chain as a functional receptor.

After 10,000 permutations, within-model BH support in the state-resolved tumor models was observed for *APP–CD74* in all three subtype models; *NCAM1–FGFR1* in all three subtype models; *SPP1–(ITGAV + ITGB1)* in the MYC and TYR models; *SPP1–CD44* in the TYR model; and *PTN–PTPRZ1* in the TYR model. For *APP–CD74*, the adjusted values were 0.00194–0.00284; corresponding values for *NCAM1–FGFR1* were 0.00194–0.00284. These values refer to the original models with separate gene-selected testing families (Figure 6A,B; Table S13B).

*APP–CD74* passed within-model Benjamini–Hochberg (BH) correction under both definitions in MYC, SHH, and TYR (q = 0.00627–0.00875). For the models including Unannotated nuclei, *APP–CD74* q values were MYC 0.00627, SHH 0.00875, and TYR 0.0068. The Jaccard overlap of tumor–glia interaction sets between definitions was 0.67–0.76 at finite *p* < 0.05 and 0.65–0.67 at BH q < 0.05. These results indicate that some candidate associations persist while the broader inferred interaction repertoire depends on tumor inclusion (Table S13A,C). *NCAM1–FGFR1* also remained BH-supported in all three subtypes under both definitions. In TYR, *SPP1–(ITGAV + ITGB1)* and *PTN–PTPRZ1* lost BH support after Unannotated inclusion; in MYC, *SPP1–CD44* gained BH support. These are changes in within-model support, not statistical tests of changes between definitions.

The earlier all-malignant comparison yielded nominal interaction-set overlap of 0.51–0.56 and BH-supported overlap of 0.49–0.50, with *APP–CD74* BH-supported in MYC and SHH. In the earlier expanded TYR analysis using CellChat’s overexpression screen, *APP* had a tumor-versus-other-groups Wilcoxon *p* = 0.0833, above the unadjusted *p* < 0.05 screening threshold; *CD74* passed in source-labeled microglia. With positive log fold change required and both ligand and receptor required in the selected gene list, *APP–CD74* was excluded before interaction testing, so no interaction *p* or q was calculated. *APP* passed the same screen in the earlier restricted TYR model (*p* = 0.0101). This screening outcome does not establish biological absence. The controlled comparison tests a common database interaction set and therefore provides an *APP–CD74* result under both definitions. These broader fits additionally changed malignant categories and eligible patient contributions, so they do not isolate Unannotated inclusion (Table S13B).

With 10,000 permutations, the MYC-2 inclusion sensitivity retained *APP–CD74* after within-model BH correction while its observed score changed from 0.0532 to 0.0520. In separate donor-specific fits, *APP–CD74* passed within-model BH correction in 7/10 eligible tumors, *NCAM1–FGFR1* in 2/3 astrocyte-eligible tumors, and *PTN–PTPRZ1* in 1/3. The corresponding nominal counts were unchanged. These counts describe recurrence of model-supported predictions across tumors, not a separate statistical test of consistency across patients. Predictions remain conditional on tumor scope, annotation, and contributing donors; within-model support does not establish functional signaling or subtype specificity (Figure 6C; Table S13).

### 3.6. Conservative Annotation Restricts Recovery of Source-Labeled Glial Cells

Of 47 source-labeled astrocytes, 23 met conservative identity criteria; expanding only within the source TME yielded 28 astrocyte candidates. The non-transporter identity set contained 14. Marker plots also demonstrated shared astroglial transcripts in malignant OPC-like and RG-like states, reinforcing the need to retain the source compartment boundary (Supplementary Figure S4). No malignant nucleus was reassigned. Across the source TME, refinement yielded 15 microglia-enriched, 16 macrophage-like, 67 BAM-like, and 173 unresolved myeloid nuclei; these operational assignments are not ontogeny measurements (Figure S4; Table 1 and accompanying annotation cell registry).

Conservative astrocyte scoring retained 12/23 homeostatic, 5/23 pan-reactive, 3/23 inflammatory, and 5/23 ECM-associated nominal calls at the two-gene threshold. These selected-set proportions should not be generalized to all astrocytes. Paired pseudobulk eligibility fell to one tumor (TYR-2; six conservative astrocytes), with a glutamate/glutamine-module difference of +1.326 log2(CPM + 1). No donor met the five-astrocyte requirement under non-transporter identity selection. Thus, the original positive median is not established as robust across independently supported astrocyte donors (Supplementary Figure S5; Table S15).

Source microglial subtype tests remained non-significant under rank scoring (minimum BH-adjusted *p* = 0.621) and removal of the detection filter (minimum adjusted *p* = 0.562). Conservative microglia could not support subtype testing: only one donor contributed at least five such nuclei. Across 75 single-gene omissions from individual programs (73 distinct genes across nine programs), the highest-relative-score label changed for 2–18 of 288 nuclei per omission (0.7–6.3%), illustrating sensitivity of that display convention without establishing a functional state (Table S15).

Increasing the astrocyte minimum from five to ten nuclei left MYC-3 and TYR-2; no donor had twenty source astrocytes. On fixed donor fits, *NCAM1–FGFR1* passed within-model BH correction in one of the two remaining eligible donors at ten, and *PTN–PTPRZ1* in one of two. No conservative astrocyte donor reached ten nuclei. At a twenty-nucleus microglial minimum, tumor-to-microglia *APP–CD74* passed within-model BH correction in five of six eligible donors. These post-fit filters quantify loss of support; they do not demonstrate robustness to refitting at higher thresholds (Table S16).

### 3.7. External Cohort Comparison

The external dataset comprised 42,703 nuclei from eight specimens and four patients. Conservative annotation retained 27,572 CNV-supported tumor candidates, 80 microglia, 60 macrophage-like cells, 525 BAM-like cells, and 671 unresolved myeloid cells. No astrocytes met the criteria; this is a failure to recover a sufficiently supported population, not evidence of biological absence. Seventy-two of 80 microglia came from one recurrent specimen (SCPCL000812). Therefore, the cohort cannot validate astrocyte findings, and microglia-specific results are largely a single-specimen observation (Supplementary Figures S7 and S8; Table S17).

Of 24 prespecified external model configurations, 17 eligible configurations were evaluated (16 distinct input cell sets) and seven were skipped because fewer than two groups or no CNV-supported tumor group met eligibility. In the primary conservative models (minimum 20 nuclei per group), tumor-to-myeloid *APP–CD74* passed within-model BH correction in at least one eligible myeloid population in 5/5 evaluable specimens, representing 4/4 evaluable patients; myeloid-to-tumor *SPP1–(ITGAV + ITGB1)* passed in 3/5 evaluable specimens, representing 2/4 evaluable patients; and myeloid-to-tumor *SPP1–CD44* passed in 1/5 evaluable specimens, representing 1/4 evaluable patients. All models used 10,000 permutations. A patient is counted once when at least one specimen supports a candidate. Broader-annotation and minimum-ten sensitivities are reported with all eligible denominators in Table S17. These counts summarize recurrence of within-model support, not a patient-level significance test. Astrocyte candidates were not assessable. Repeated computational support does not establish receptor activation or confirm a microglia-specific mechanism (Figure S8; Table S17).

At the independent-patient level, pooled operational myeloid *CD74* and antigen-presentation-associated expression showed a positive but non-significant association (rho = 0.800; exact two-sided *p* = 0.333; BH-adjusted *p* = 0.750; four patients). BAM-like analysis included three patients; microglia and macrophage-like groups each had one patient meeting the twenty-cell requirement and could not support patient-level association tests. The discovery *CD74* result could not be statistically confirmed in this small external cohort. With four patients, the minimum attainable exact two-sided Spearman *p* value is 2/24 = 0.083, even under perfect rank correlation; this analysis cannot meet a 0.05 threshold and does not provide evidence against the discovery association. Different myeloid mixtures, recurrent-disease sampling, and platform differences further limit direct comparison. Molecular-subtype validation was impossible because subgroup labels were unavailable.

## 4. Discussion

### 4.1. Glial Populations Among Recovered Nonmalignant Nuclei

This focused reanalysis describes glial expression programs within a small recovered nonmalignant compartment. Only 516 of 17,564 nuclei (2.94%) were classified as TME, including 47 astrocytes across 12 tumors and 288 source-labeled microglial nuclei. The latter were the largest recovered TME population, but the source label does not establish resident origin or distinguish all macrophage-like states. Cellular composition varied substantially among tumors and should not be generalized to the intact ATRT microenvironment (Figure 2). Prior reports of subgroup-dependent lymphoid and myeloid infiltration in ATRT [6,7], pediatric brain-tumor immune heterogeneity [10], and *SPP1*-high macrophage populations in extracranial rhabdoid tumors [41] provide context without validating these particular populations or their proposed functions.

The deposited annotations did not separately resolve adaptive lymphocyte populations; therefore, the recovered cellular composition should not be interpreted as a complete representation of the immune landscape. Consequently, the present data could not establish whether ATRT is immunologically “hot” or “cold,” as immune contexture depends on multiple complementary measures—including lymphocyte infiltration, spatial organization, and functional immune activity [42]—that extend beyond single-nucleus transcriptomic composition.

Retaining the source malignant and nonmalignant assignments preserves comparability with the atlas. Cell2Sentence agreed with 72.9% of broad-lineage assignments and approximately 64% of astrocyte assignments (Supplementary Figure S1A). Because Cell2Sentence uses the same expression data, this is an annotation-method comparison, not an independent assessment or biological validation. Agreement cannot establish cell identity, and disagreement remains a source of uncertainty in this small cohort.

Compared with the original atlas, which emphasized malignant differentiation trajectories [2], the added contribution is the donor-resolved examination of nonmalignant expression, explicit annotation sensitivities, exact receptor-complex checks, and external-cohort comparison. Several results lose support under conservative restrictions. Reporting this attrition narrows the claims and identifies the populations for which additional sampling is most needed.

### 4.2. Astrocyte Programs and Annotation-Dependent Support

Only 12/47 source-labeled astrocytes met the nominal homeostatic-program criterion, whereas 23/47 were below all four program thresholds. These counts do not support describing the population as predominantly homeostatic. Cells below all four thresholds did not show sufficient expression enrichment to meet the specified program criteria, potentially reflecting sparse transcript detection or weaker expression of the selected genes. They may also express programs not captured by these panels, but the present analysis cannot distinguish these possibilities. The four overlapping programs and variable gene detection instead indicate heterogeneous expression among a small recovered population (Figure 3). Their descriptive character is consistent with recommendations to avoid assigning a single functional identity from reactive-astrocyte transcription alone [11].

*SLC1A2*, *SLC1A3*, and *GLUL* expression motivates a hypothesis concerning glutamate handling and glutamine synthesis, but nuclear RNA does not measure extracellular glutamate clearance, metabolic flux, or transfer of glutamine to tumor cells. The paired module result varied by donor: ATRT-TYR-2 and ATRT-MYC-3 had positive differences, whereas ATRT-SHH-4 had a negative difference. With only three eligible tumors and no matched normal-brain astrocyte reference, the analysis cannot establish a general ATRT-associated increase or tumor-induced reprogramming. Glutamine-synthetase-dependent growth in glioblastoma [19] and sensitivity of ATRT-MYC models to glutamine antagonism [43] provide reasons to test this hypothesis experimentally, without establishing an astrocytic nutrient source in ATRT. Likewise, extracellular glutamate release and glutamatergic inputs reported in glioma [44,45] do not demonstrate analogous processes here.

Selection of astrocytes using glutamate transporters can enrich the same expression program subsequently tested. The non-transporter identity sensitivity addresses this circularity but leaves no eligible matched donor. The current data therefore cannot separate general astroglial identity, selection effects, and ATRT-associated metabolic adaptation.

### 4.3. Astrocyte Ligand–Receptor Hypotheses

The initial pooled model nominated *NCAM1–FGFR1* and *PTN–PTPRZ1* among astrocyte-to-tumor candidates (Figure 4D). Fresh same-donor fits retained them nominally in two of three and one of three eligible tumors, respectively. Fewer donors met conservative annotation and higher cell-count requirements, limiting assessment of consistency across tumors. Experimental evidence concerning tenascin-C, PDGF-C, and FGF receptor biology [46,47,48,49,50] offers context for testing related candidates.

Our prior study found that induced-pluripotent-stem-cell-derived astrocytes altered cisplatin sensitivity in ATRT cell models [51]. That experiment supports investigating astrocyte–tumor relationships but did not test the specific ligand–receptor candidates identified here. Evidence for gap-junction effects in brain metastasis [52], NCAM1–FGFR1 receptor trafficking [53], PTN–PTPRZ1 biology in glioblastoma [54], and astrocyte effects in other CNS tumor models [18,55] likewise provides external mechanistic context. These precedents should not be interpreted as functional validation of the present transcriptomic predictions.

### 4.4. Microglial Identity and Overlapping Expression Programs

The source microglial group exhibited overlapping inflammatory-associated, immunoregulatory-associated, phagocytic/lipid-associated, antigen-presentation-associated, and other programs (Figure 5). Continuous scores and single-gene-omission sensitivity make their dependence on gene-set composition explicit. Conservative annotation identified only a small microglia-enriched subset, alongside macrophage-like, BAM-like, and unresolved profiles. Thus, pooled state-associated expression should not be attributed exclusively to resident microglia. Prior glioma and macrophage studies describe related continua and context dependence [22,23,27,28,30,31,56,57,58].

Cell-type annotation remains a context-dependent challenge in transcriptomic analysis. Assignments can vary with the computational method, marker selection, reference dataset, and level of cellular detail being resolved. Benchmarking studies demonstrate that annotation performance differs across datasets and classification tasks, particularly when closely related populations or populations absent from the reference are encountered [59]. In brain tumors, distinguishing microglia from other macrophage populations additionally requires consideration of lineage-associated signatures alongside overlapping activation programs [28]. These considerations motivated our use of published labels for primary analyses and composite-marker refinement for sensitivity analyses restricted to the nonmalignant compartment. Conservative criteria prioritize coherent identity-marker evidence but may exclude cells with incomplete marker detection. We therefore treat conservative and broader populations as complementary analytical definitions, rather than assuming that either provides definitive cell identities. This framework makes annotation uncertainty explicit and identifies which findings remain supported across alternative population definitions.

Expression of *SPP1*, *APOE*, *APOC1*, and *GPNMB* was consistent with phagocytic/lipid-associated and tumor-associated myeloid transcriptional features, while *CD74*, *B2M*, and *HLA-DRA* supported an antigen-presentation-associated expression program. These terms describe gene sets, not measured phagocytosis, lipid flux, antigen presentation, or immune suppression. *SPP1*-high macrophages have also been reported in extracranial malignant rhabdoid tumors [41], providing disease-family context while underscoring that these transcripts do not establish resident microglial origin. Coexisting programs motivate testing of multiple myeloid functions without assigning a single biological role.

### 4.5. Candidate Microglial and Tumor Interactions

*SPP1–CD44* and *SPP1–(ITGAV + ITGB1)* are exact ligand–receptor candidates, not single integrin-chain interactions (Figure 6). Their within-donor retention and sensitivity to tumor definition are more informative than pooled subtype probabilities alone. Prior experiments link SPP1-related biology to glioma and other tumor systems [31,32,33,60,61,62,63], and *SPP1* expression has been reported in ATRT models [64]. These observations motivate experiments but do not show SPP1 secretion, receptor activation, a feedback loop, or a causal effect in the analyzed tumors.

*APP–CD74* was repeatedly nominated in the fresh subtype and same-donor models (Figure 6B,C). Its interpretation is informed by the new donor-level analysis: *CD74* expression tracked an antigen-presentation score from which *CD74* itself was excluded, whereas its immunoregulatory association did not remain significant after correction across nine modules. Neither result shows that APP caused these programs or activated CD74. APP/CD74-related findings in glioblastoma and other tumors [65,66,67], MIF/CD74 biology [68,69], TGF-beta-associated glioma phenotypes [70], and GAS6/TAM-receptor functions [71,72] provide pathway-specific hypotheses for ATRT experiments. Differences between subtype objects remain qualitative because patient composition, cell recovery, and model selection can affect the inferred probabilities.

Cross-tumor functional studies provide pathway-specific context. In gastric cancer, APP–CD74 blockade altered tumor-associated macrophage polarization and enhanced response to PD-1 inhibition [73]. In glioma, brain metastasis, and medulloblastoma models, MIF–CD74 signaling altered CD74-positive brain myeloid or tumor-associated immune states [68,74,75], and an accompanying commentary highlighted the potential brain-specific relevance of this pathway [76].

The external cohort provides additional transcriptomic evidence for selected candidates; their functional significance requires experimental assessment. Repeated specimens belong to four patients, and most conservative microglia derive from one specimen. The *CD74*–antigen-presentation association was positive but not significant across these four patients, whose number precludes an exact two-sided *p* < 0.05 even under perfect correlation, and differences in myeloid composition and sampling limit direct comparison. A repeated ligand–receptor nomination is evidence of computational recurrence under stated settings, not validation of a reciprocal mechanism. Whether immune-associated effects induce malignant-state transitions in ATRT, as described in experimental glioblastoma contexts [77], remains unknown.

### 4.6. A Conceptual Model of the Recovered Tumor Microenvironment

**Figure 7:**
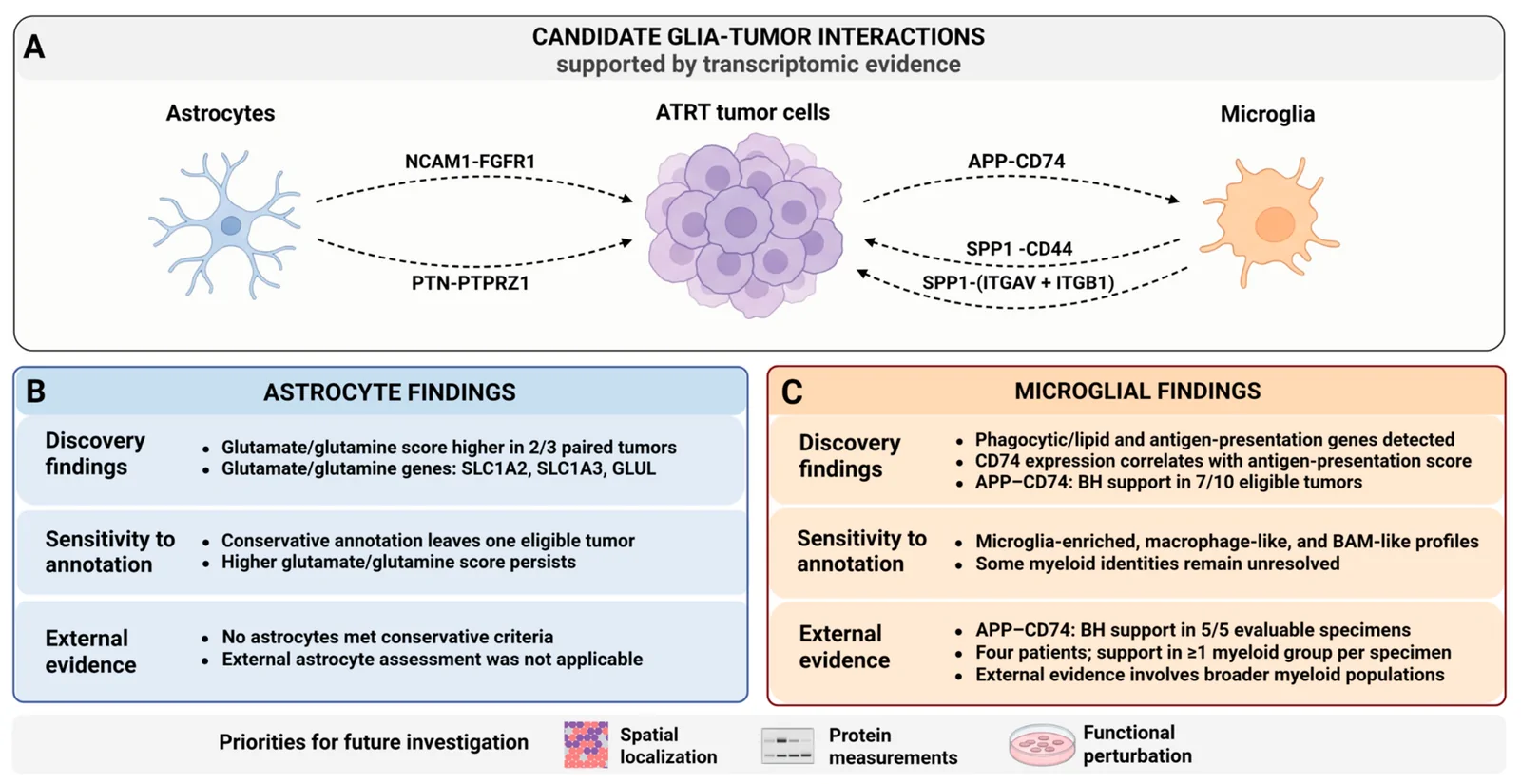
Glial transcriptional programs and candidate interactions in the ATRT microenvironment. (**A**) Selected expression-supported ligand–receptor candidates linking astrocytes, microglia, and tumor cells. Dashed arrows indicate the directions modeled by CellChat and represent hypotheses requiring experimental validation. *ITGAV and ITGB1* constitute the modeled integrin receptor complex. (**B**) Astrocyte findings in the discovery cohort, sensitivity to conservative annotation, and external assessment. Glutamate/glutamine-module scores were higher in astrocytes than in matched tumor cells in two of three evaluable tumors. This difference persisted in TYR-2, the single tumor eligible under conservative annotation. No astrocytes met conservative criteria in the external cohort, preventing external assessment. (**C**) Microglial expression programs, annotation sensitivity, and external support for selected interactions. Across 12 discovery tumors, *CD74* expression correlated with the antigen-presentation module score calculated without *CD74* (Spearman ρ = 0.923; BH-adjusted *p* = 0.00045). Tumor-to-microglia *APP–CD74* received within-model BH support in seven of ten eligible discovery tumors. In the external cohort, tumor-to-myeloid *APP–CD74* received support in at least one eligible myeloid population in each of five evaluable specimens from four patients. These findings provide external myeloid-level support without establishing a microglia-specific mechanism. Primary discovery analyses used published cell labels; marker refinement was restricted to the nonmalignant compartment. Macrophage-like and border-associated macrophage (BAM)-like labels describe expression profiles rather than proven origin. Only one discovery donor contributed at least five conservatively identified microglia, limiting assessment of consistency across patients within that subset. CellChat models used 10,000 permutations; BH support denotes within-model Benjamini–Hochberg-adjusted q < 0.05. Spatial localization, protein measurements, and functional perturbation are priorities for testing the biological significance of these candidates.

Figure 7 summarizes the observed astrocyte and microglial gene-expression patterns and uses dashed arrows to indicate candidate glia–tumor interactions inferred from ligand–receptor analysis. It highlights the limited astrocyte donor support and distinguishes source microglial labels from conservative identity assignments. The diagram does not infer complementary physiological functions, nutrient support, or reciprocal signaling.

The candidate links span developmental, immune-related, and matrix-associated gene-expression themes. They provide a framework for choosing validation experiments, but pooling nuclei or detecting ligand and receptor transcripts does not show that the corresponding cells are adjacent or communicate in an individual tumor. In particular, a candidate retained in a subtype object cannot be assigned to that subtype independently of its contributing donors.

The diagram provides a way to select experiments after accounting for donor support and annotation uncertainty. It cannot determine whether a candidate promotes or restrains tumor growth, or whether ATRT has reprogrammed adjacent glia. Such conclusions require appropriate reference tissue and direct experimental tests.

### 4.7. Future Directions

The present study provides an exploratory framework for investigating glia–tumor interactions in ATRT. An important next step will be to integrate transcriptomic findings with spatially resolved approaches, including multiplex immunofluorescence, RNAscope, and spatial transcriptomics, to define the localization and interactions of astrocytes, microglia, and ATRT cells within intact tumor tissue. Functional coculture and organoid models will further distinguish soluble, contact-dependent, and reciprocal signaling mechanisms that influence tumor growth, lineage state, therapeutic response, and microglial function.

Candidate pairs can guide spatial colocalization and receptor-complex protein measurements before perturbation in appropriate models. Stable-isotope tracing would be needed to test the proposed glutamate/glutamine connection. Myeloid assays could examine phagocytic and antigen-presentation hypotheses, with checkpoint experiments informed by work in other tumor settings [78].

### 4.8. Study Limitations

The discovery TME comprised only 516 nuclei, including 47 source-labeled astrocytes from 12 tumors. Paired analyses used 29 astrocytes from three tumors; conservative identity criteria left one eligible tumor, and the non-transporter identity sensitivity left none. Each astrocyte-qualified subtype had only one contributing donor. Consequently, nuclei from the same patient are not independent patient-level observations, subtype effects cannot be separated from astrocyte donor effects, and the positive glutamate-module median cannot be generalized. Conservative markers increase specificity at the cost of sensitivity, particularly when activation or nuclear dropout reduces homeostatic transcripts.

The external cohort contained only four patients and no accepted astrocyte population; most accepted microglia came from one specimen. It lacks supplied MYC/SHH/TYR labels and therefore cannot validate astrocyte findings or molecular-subtype differences. Absence of an accepted population is not proof of biological absence. RNA-derived CNV supports an operational tumor group but is not orthogonal DNA confirmation. Neither cohort includes matched normal astrocytes suitable for attributing expression to ATRT-specific reprogramming. CellChat results depend on database, group definition, cell recovery, and pooling; pooled group-label permutations are not blocked by donor and do not test patient-level recurrence. Increasing the number of permutations does not increase the number of independent patients studied. The data do not measure protein abundance, secretion, receptor activation, spatial proximity, metabolic flux, or causal function. Source-model execution details were incompletely retained for the initial pooled CellChat and Cell2Sentence outputs; fresh analyses are separately versioned and fully archived.

## 5. Conclusions

This focused reanalysis extends the published ATRT atlas by examining astrocyte and microglial transcriptional programs and expression-supported candidate interactions with tumor cells. Astrocytes displayed heterogeneous programs and expressed genes involved in glutamate handling and glutamine synthesis, including *SLC1A2*, *SLC1A3*, and *GLUL*. Glutamate/glutamine-module scores were higher in astrocytes than in matched tumor cells in two of three evaluable tumors, motivating further investigation of astrocyte metabolic biology in ATRT. Microglial programs overlapped, and *CD74* expression correlated strongly with an antigen-presentation module calculated without *CD74*, placing this candidate receptor within a defined immune-related transcriptional context. *APP–CD74* emerged as a priority interaction candidate, receiving within-model BH support in seven of ten eligible discovery tumors and in at least one myeloid population in each of five evaluable external specimens from four patients. *NCAM1–FGFR1*, *PTN–PTPRZ1*, and SPP1-related interactions provide additional candidates for investigation. External support concerned broader myeloid populations, while astrocyte findings remained limited to the discovery cohort. Conservative astrocyte annotation preserved the glutamate/glutamine difference in the single eligible tumor, highlighting the need to establish its consistency across patients. Together, these findings prioritize astrocyte glutamate/glutamine-related programs and candidate tumor–myeloid interactions as specific directions for studying the ATRT microenvironment. Larger cohorts, spatial and protein-level measurements, and functional perturbation studies are needed to determine whether these transcriptional observations reflect biological communication and contribute to tumor behavior.

## Figures and Tables

**Table 2:** Gene sets used to evaluate astrocyte transcriptional programs.

State Program	Genes used for Scoring	Representative Genes in Main Figure	Sources
Homeostatic	*ALDH1L1*, *AQP4*, *SLC1A2*, *SLC1A3*, *GLUL*, *GJA1*, *S100B*, *SOX9*, *FGFR3*, *ATP1A2*, *ATP1B2*, *KCNJ10*, *SLC4A4*, *GPC5*	*SLC1A2*, *SLC1A3*, *AQP4*	[11,12,13]
Pan-reactive	*GFAP*, *VIM*, *CD44*, *SERPINA3*, *LCN2*, *CHI3L1*, *OSMR*, *STAT3*, *SOCS3*, *JUN*, *FOS*, *CEBPB*, *ICAM1*, *VCAM1*, *TIMP1*, *ANXA1*	*GFAP*, *VIM*, *CD44*	[11,16]
Inflammatory	*C3*, *SERPING1*, *GBP2*, *MX1*, *IFITM3*, *STAT1*, *CXCL10*, *CCL2*, *IL1B*, *TNF*, *NFKBIA*, *IRF1*, *HLA-DRA*, *HLA-DRB1*, *CD74*, *IFIT1*, *IFIT3*, *ISG15*, *CFB*, *C1S*, *C1R*	*STAT1*, *IFIT3*, *C1S*	[11,16,17]
ECM-remodeling	*S100A10*, *PTX3*, *EMP1*, *SPHK1*, *CD109*, *CLCF1*, *TGM1*, *TM4SF1*, *SPARCL1*, *CLU*, *APOE*, *THBS1*, *VEGFA*	*SPARCL1*, *EMP1*, *VEGFA*	[9,11,16]

**Table 3:** Gene sets used to evaluate source microglial transcriptional programs.

State Program	Genes Retained for Scoring (≥9 Nuclei)	Representative Genes in Main Figure	Sources
Homeostatic	*P2RY12*, *TMEM119*, *SALL1*, *HEXB*, *CX3CR1*, *GPR34*, *SLC2A5*, *BIN1*, *CSF1R*, *TREM2*	*CSF1R*, *SLC2A5*	[24,30]
Inflammatory	*IL1B*, *TNF*, *CCL2*, *CCL3*, *CCL4*, *NFKBIA*, *STAT1*, *IRF1*, *CD86*	*CD86*, *STAT1*	[22,23,30]
Immunoregulatory	*MRC1*, *CD163*, *MSR1*, *IL10*, *TGFB1*, *MAFB*, *VSIG4*, *FOLR2*	*MSR1*, *CD163*	[22,23,30]
Interferon-response	*ISG15*, *IFIT1*, *IFIT2*, *IFIT3*, *MX1*, *OAS1*, *OAS2*, *GBP1*, *STAT1*, *IRF7*	*STAT1*, *MX1*	[27,28,30]
Phagocytic and lipid processing	*APOE*, *APOC1*, *LPL*, *SPP1*, *GPNMB*, *TREM2*, *CTSD*, *CTSB*, *LGALS3*, *FABP5*, *ABCA1*	*CTSB*, *ABCA1*	[25,26,27,28,30]
Antigen-presentation	*HLA-DRA*, *HLA-DRB1*, *HLA-DPA1*, *HLA-DPB1*, *CD74*, *CIITA*, *B2M*, *TAP1*	*B2M*, *CD74*	[27,28,30]
Hypoxia and stress-response	*HIF1A*, *VEGFA*, *LDHA*, *BNIP3*, *NDRG1*, *SLC2A1*, *HMOX1*	*HIF1A*, *LDHA*	[27,28,30]
Complement	*C1QA*, *C1QB*, *C1QC*, *C3*, *CFB*, *SERPING1*	*C1QB*, *C3*	[25,26,27,28,30]
Proliferation-associated	*MKI67*, *TOP2A*, *CENPF*, *UBE2C*, *TYMS*, *PCNA*	*TOP2A*, *CENPF*	[27,28,30]

## Data Availability

GSE283839 data are available from GEO. External processed ATRT data are available through ScPCA project SCPCP000010 (https://scpca.alexslemonade.org/projects/SCPCP000010 (accessed on 29 September 2026)), contributed by Arash Nabbi and Trevor J. Pugh. The original study code repository is https://github.com/harryxue3-commits/ATRT-TME-snRNAseq-analysis (accessed on 22 October 2026). The accompanying revision package adds cell-level inclusion files, patient/sample labels, frozen annotation rules, exact database identities, reproducible scripts, and tabular model outputs. Saved CellChat objects and normalized input provenance are retained with the local analysis archive. Newly generated files have not been assumed to be present in the existing public repository.

## Supplementary Materials

The following supporting information can be downloaded at: https://www.mdpi.com/article/10.3390/cancers18213455/s1, Figure S1: Annotation comparison; Figure S2: Ranked-gene marker evidence; Figure S3: Astrocyte thresholds and donor-level *CD74* associations; Figure S4: Marker assessment; Figure S5: Annota-tion sensitivity; Figure S6: Ligand and receptor expression within individual tumors; Figure S7: External cohort expression; Figure S8: External CellChat candidates; Table S1: Cell-Type Counts and Proportions; Table S2: Astrocyte and Microglia State Marker Tables; Table S3: Astrocyte Support Module Genes; Table S4: CellChat Ligand–Receptor Panel; Table S5: Pooled Astro-cyte-to-Tumor CellChat Interactions; Table S6: Cell2Sentence Annotation Comparison and Rank-Weighted Scores; Table S7: Gene Markers and Abbreviations; Table S8: Astrocyte threshold sensitivity; Table S9: Sample inclusion and provenance; Table S10: Microglial gene filter audit; Table S11: *CD74* associations; Table S12: Paired astrocyte comparisons; Table S13: CellChat sen-sitivity to tumor inclusion; Table S14: Clinical metadata; Table S15: Annotation and scoring sen-sitivities; Table S16: Donor support and receptor definition; Table S17: External cohort analyses; Table S18: Cell2Sentence implementation audit.

## Abbreviations

The following abbreviations are used in this manuscript:

ATRT	Atypical teratoid/rhabdoid tumor
BAM	Border-associated macrophage
BH	Benjamini–Hochberg
C2S	Cell2Sentence
CNS	Central nervous system
CNV	Copy-number variation
CP10K	Counts per 10,000
CPM	Counts per million
ECM	Extracellular matrix
GBM	Glioblastoma
GEO	Gene Expression Omnibus
IPC	Neuronal intermediate precursor cell
NPC	Neuronal precursor cell
OPC	Oligodendrocyte precursor cell
QC	Quality control
RG	Radial glia
ScPCA	Single-Cell Pediatric Cancer Atlas
snRNA-seq	Single-nucleus RNA sequencing
TME	Tumor microenvironment
UMAP	Uniform manifold approximation and projection
