# Supplementary material for: Astrocyte and Microglial Transcriptional Programs in the Atypical Teratoid/Rhabdoid Tumor Microenvironment
Source: Cancers (Basel). 2026 Oct 26;18(21):3455. doi: 10.3390/cancers18213455 (PMC13762015; doi:10.3390/cancers18213455)
Supplement: Supplementary file 1 [file cancers-18-03455-s001.zip › cancers-4567692-supplementary-round1.pdf]

# Supplementary Materials for Astrocyte and Microglial Transcriptional Programs in the Atypical Teratoid/Rhabdoid Tumor Microenvironment

Supplementary Figures S1–S8 and Tables S8–S18

These supplementary materials describe astrocyte and microglial expression programs, candidate ligand–receptor associations, and sensitivity analyses in GSE283839, with an external ScPCA comparison comprising eight specimens from four patients. Primary discovery analyses retain the published cell labels and malignant assignments. Conservative annotation is used to assess sensitivity within the nonmalignant compartment. The tables report cohort composition, donor eligibility, scoring methods, and model-specific statistical support.

Figures are presented first, followed by tables in numerical order. Tables S1–S7 are supplied separately. Annotation marker panels are presented in main-text Table 1. References to manuscript figures and tables use the current numbering; archived data filenames are mapped to that numbering at the end of this document.

## Supplementary Figure S1 Annotation comparison

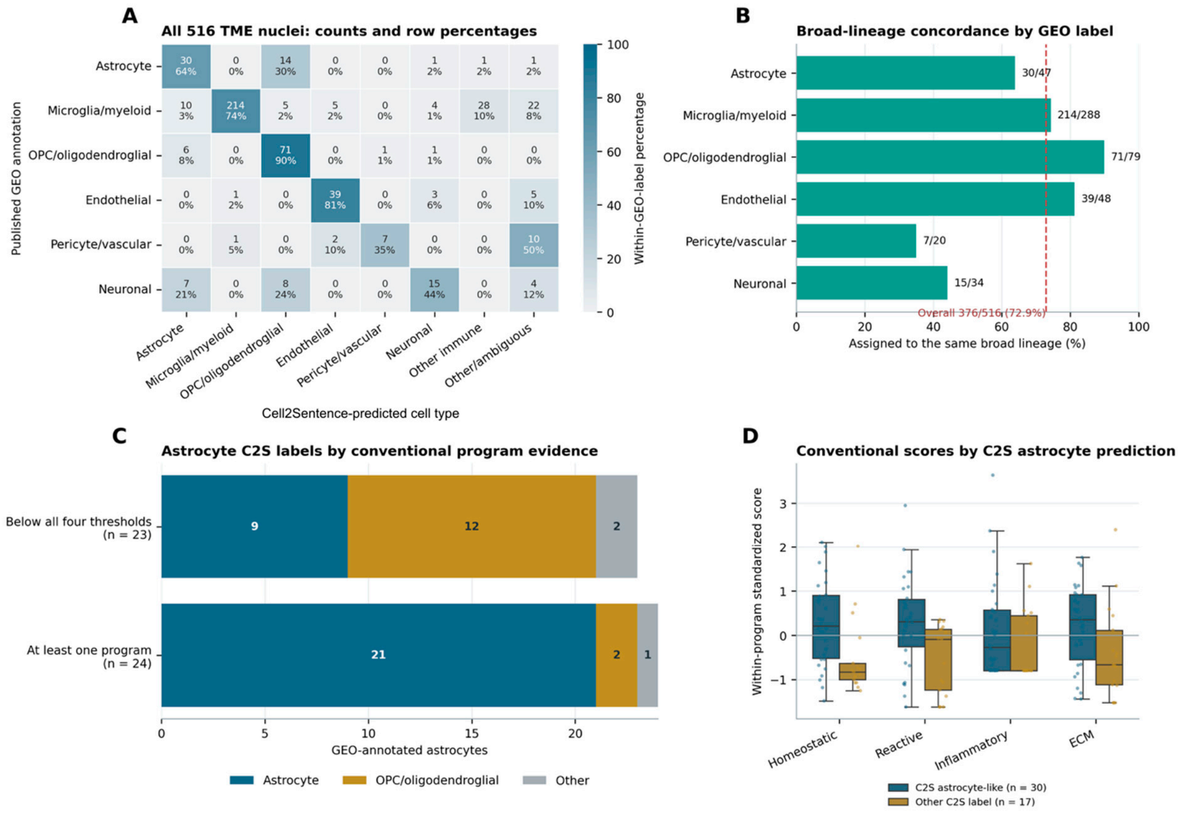


(A) Broad-lineage agreement between Cell2Sentence and GEO across 516 source TME nuclei; entries are counts and within-GEO-label percentages. (B) Concordance by source lineage; the dashed line marks overall agreement, 376/516 (72.9%). (C) Among 47 source astrocytes, Cell2Sentence assigned astrocyte-like labels to 9/23 below all four conventional program thresholds and 21/24 meeting at least one threshold. (D) Full-matrix rank scores, standardized within each program across the 47 astrocytes, grouped by Cell2Sentence astrocyte-like versus other labels. These same-data comparisons are descriptive; no inferential testing was performed. GEO labels are a reference framework, not independent ground truth.

## Supplementary Figure S2 Ranked-gene marker evidence

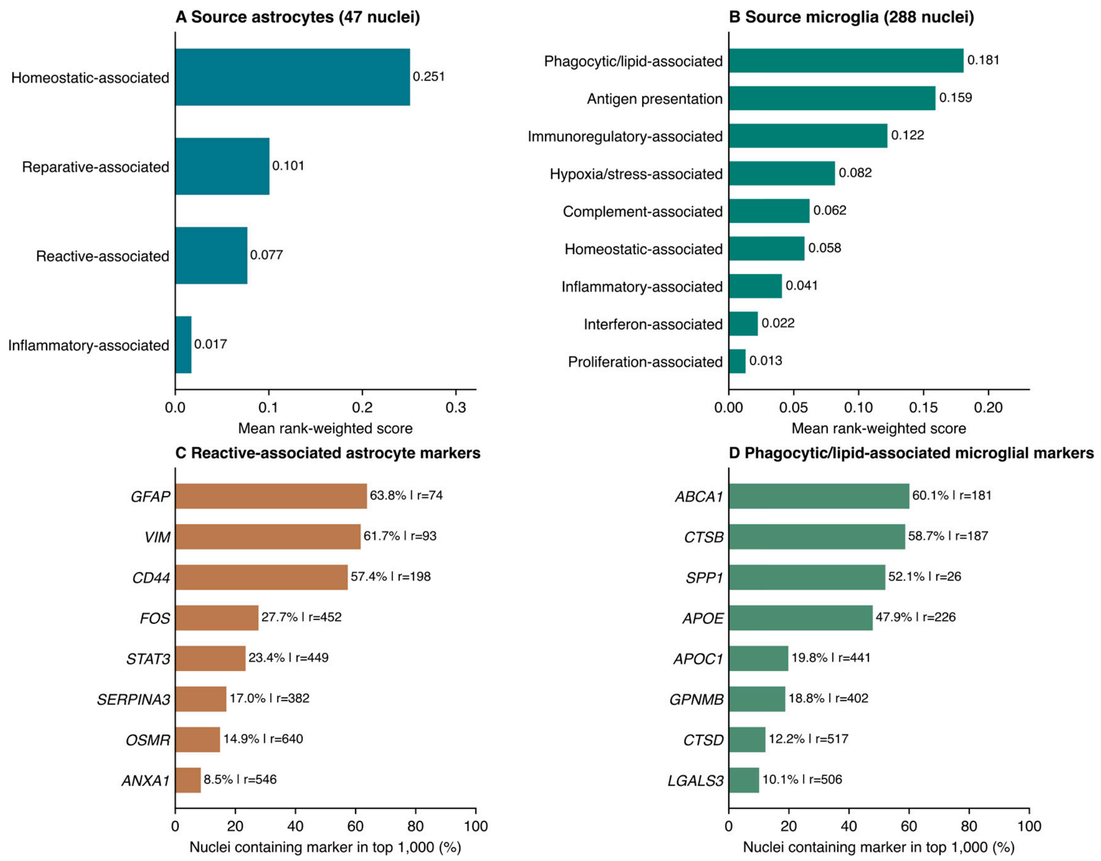


(A,B) Pooled mean rank-weighted marker evidence in 47 source astrocytes and 288 source microglial nuclei. Gene weight is (1,001 minus rank)/1,000 for a marker in the top 1,000 genes, otherwise zero; each score is the mean over the entire specified panel. All thirteen source program means were reconstructed from saved cell sentences and matched exactly at the original five-decimal precision. (C,D) Representative marker frequency within the ranked lists; r is median rank among positive nuclei. These are descriptive expression-associated programs, not functional states or proof of cellular origin. The original panel definitions are retained, including low-detection genes; this supplementary rank analysis differs from the filtered full-matrix primary scoring. The S2 rank-program gene CSV and archived source code define these supplementary panels. Program names describe expression patterns without assigning M1/M2 polarization or neurotoxicity. Source data and the reconstruction audit accompany Table S2.

**Supplementary Figure S3 Astrocyte thresholds and donor-level CD74 associations**

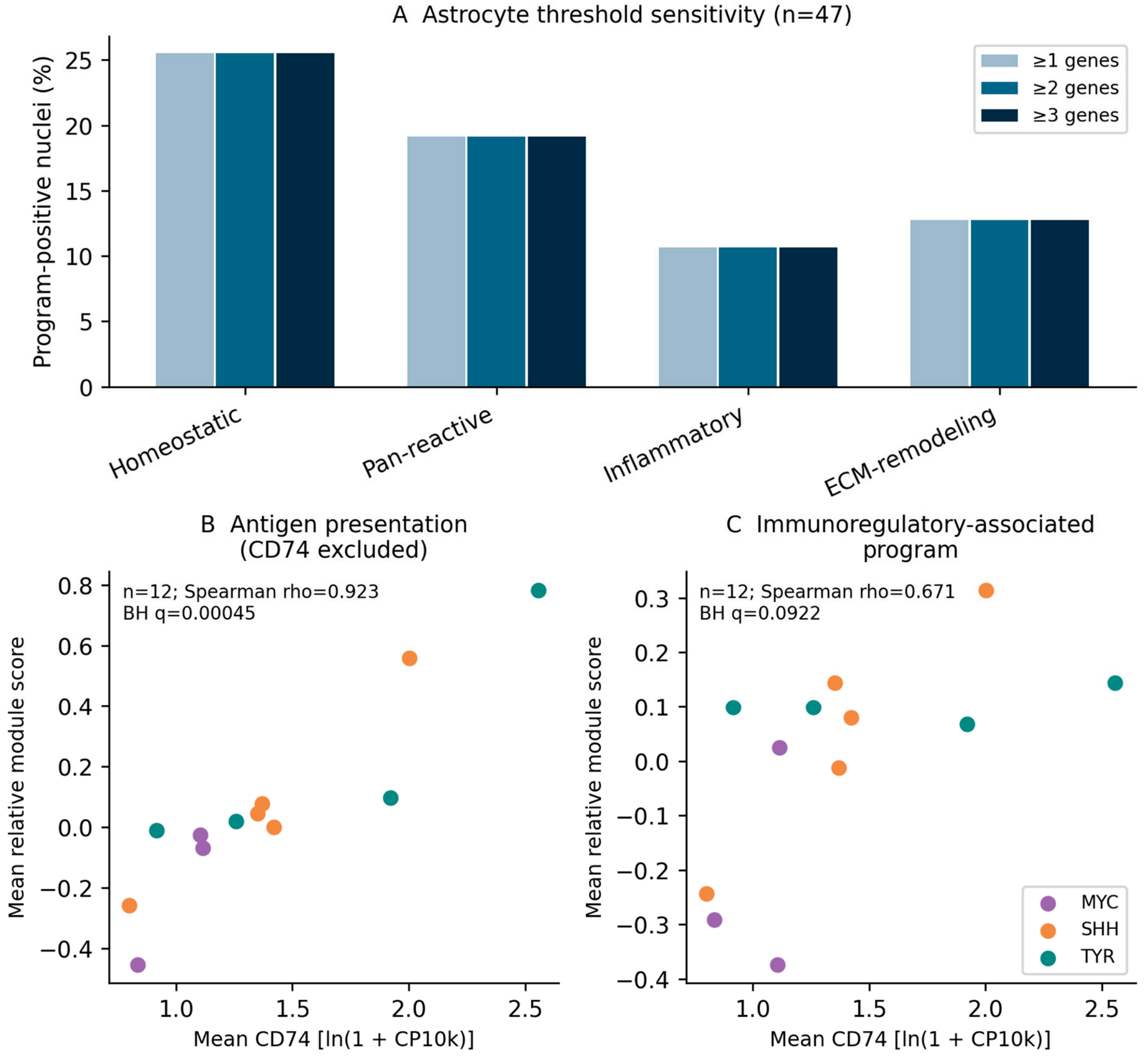


Astrocyte detection-threshold sensitivity and donor-level CD74 associations. (A) One-, two-, and three-gene detection requirements give identical proportions among 47 source-labeled astrocytes when the original matched-null cutoffs are fixed. (B,C) Each point is one tumor (n = 12), colored by molecular subtype. CD74 was removed from the antigen-presentation gene set before calculating its score. P values use 99,999 two-sided donor permutations; BH correction covers nine modules. Correlation does not establish receptor activation or a response to APP.

## Supplementary Figure S4 Marker assessment

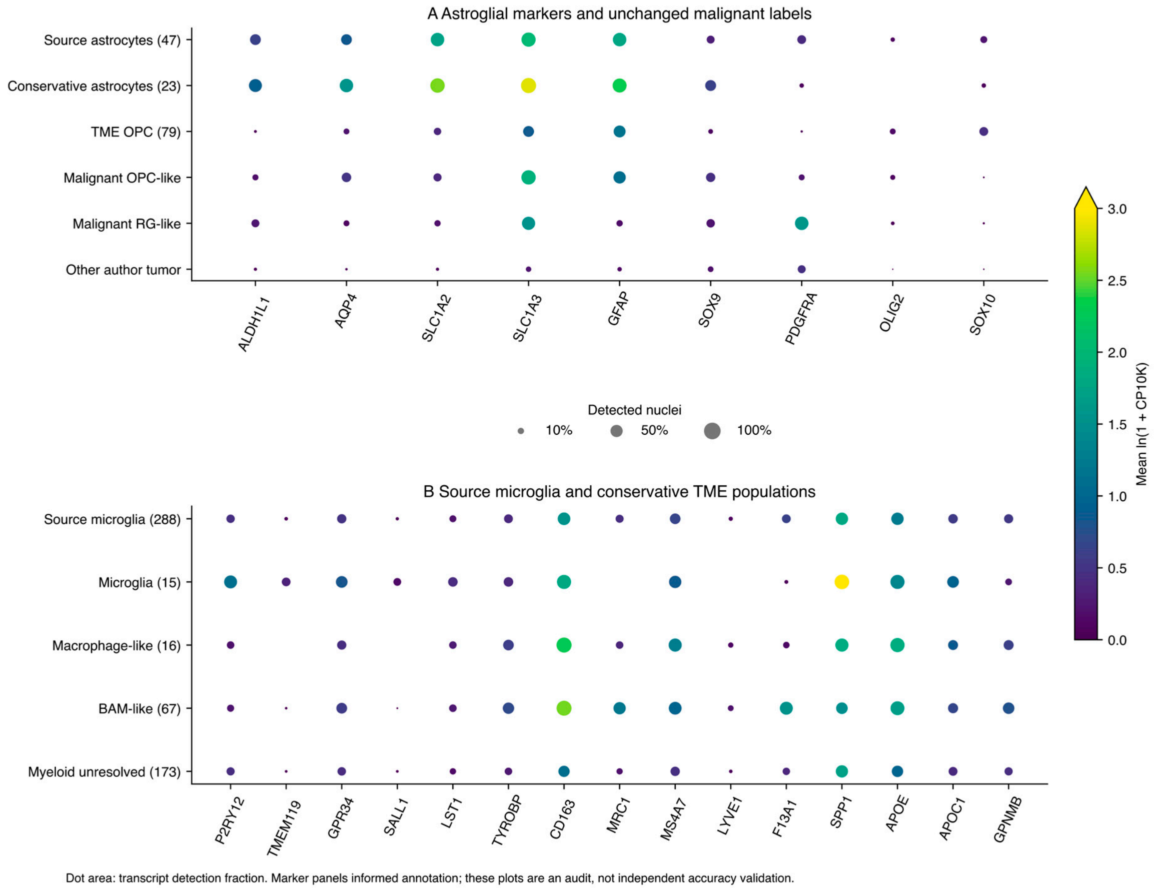


(A) Astroglial marker expression in source and conservative astrocytes, source TME OPCs, and unchanged malignant groups. (B) Myeloid markers across source and refined groups. Dot area shows the fraction with at least one transcript; color is pooled mean ln(1 + CP10K). No dot means zero detection. BAM denotes border-associated macrophage. BAM-like and macrophage-like labels describe expression profiles and do not establish anatomical location or blood-derived origin. The panels were used for annotation and therefore do not provide independent accuracy validation. Marker panels and anchor requirements are summarized in main-text Table 1. Exact counts, cell IDs, and plotted values are retained in the accompanying annotation archive.

## Supplementary Figure S5 Annotation sensitivity

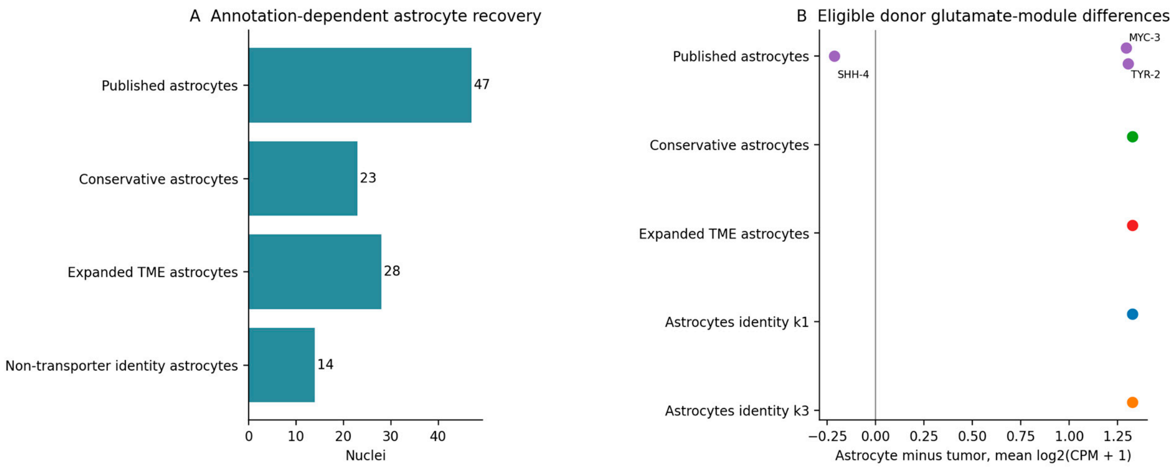


(A) Astrocyte recovery under four overlapping definitions. (B) Eligible donor-level astrocyte-minus-tumor glutamate/glutamine module differences. Each point represents a donor meeting the minimum of five astrocyte and 20 state-resolved tumor nuclei; jitter separates nearby values and has no analytical meaning. Conservative identity leaves only TYR-2 eligible, and non-transporter identity leaves no eligible donor. Identity k1 and k3 require one or three detected identity-panel genes, respectively. Non-transporter identity excludes SLC1A2 and SLC1A3 from the identity panel to assess dependence on glutamate-transporter markers. Full values, eligibility, program calls, rank-score comparisons, and single-gene omission results are in Table S15.

**Supplementary Figure S6 Ligand and receptor expression within individual tumors**

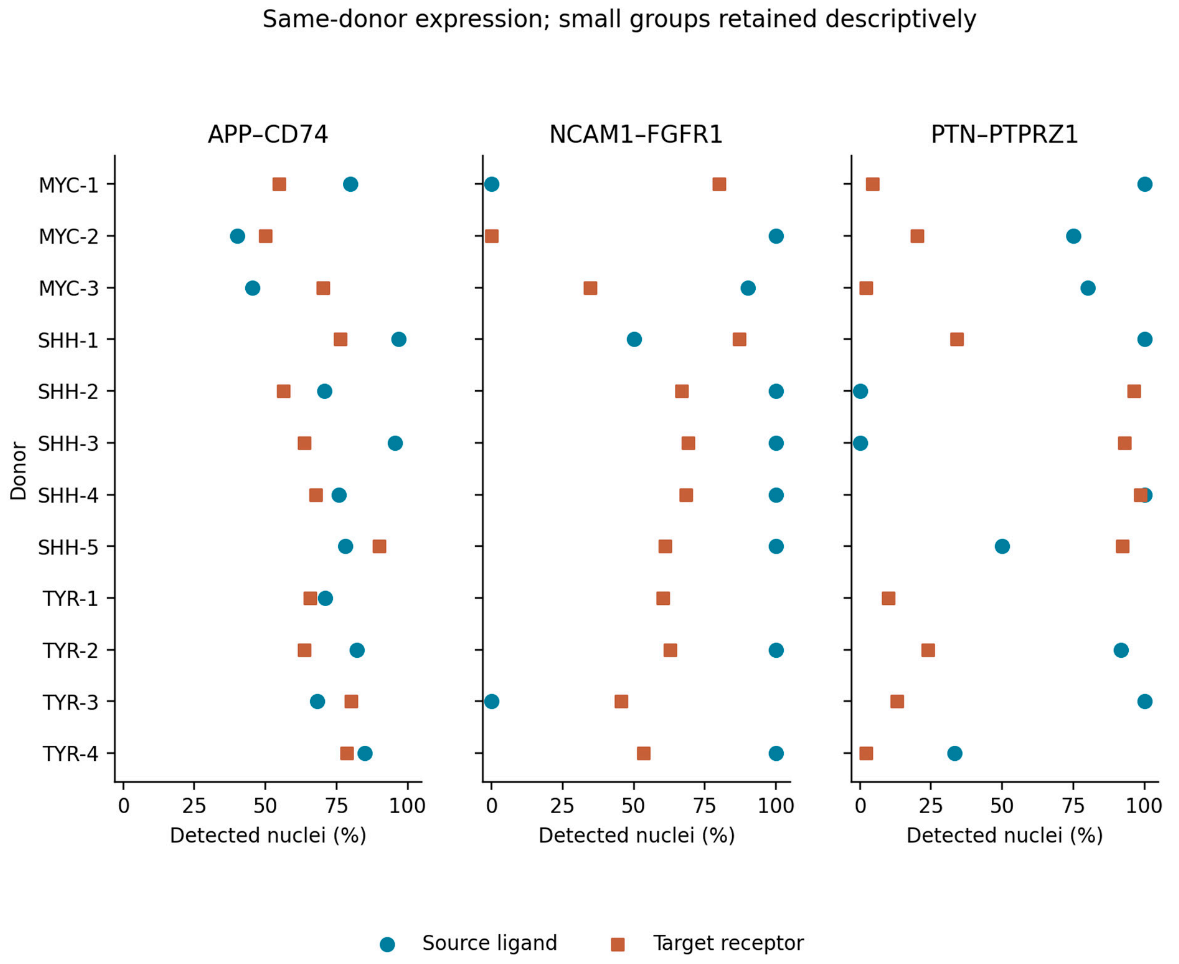


Rows align the same twelve donors across panels; source and target nucleus counts are provided in Tables S9 and S16. APP–CD74 is tumor-to-microglia; NCAM1–FGFR1 and PTN–PTPRZ1 are source-astrocyte-to-tumor. Positive detection means at least one raw transcript. Small groups are retained descriptively and do not imply CellChat eligibility. The missing ligand point for TYR-1 reflects zero recovered source astrocytes and unassessable ligand expression; its tumor receptor expression is still shown. Table S16 also supplies complete-receptor co-detection for multimeric pairs. RNA detection does not establish an assembled receptor, spatial contact, or activity.

**Supplementary Figure S7 External cohort expression**

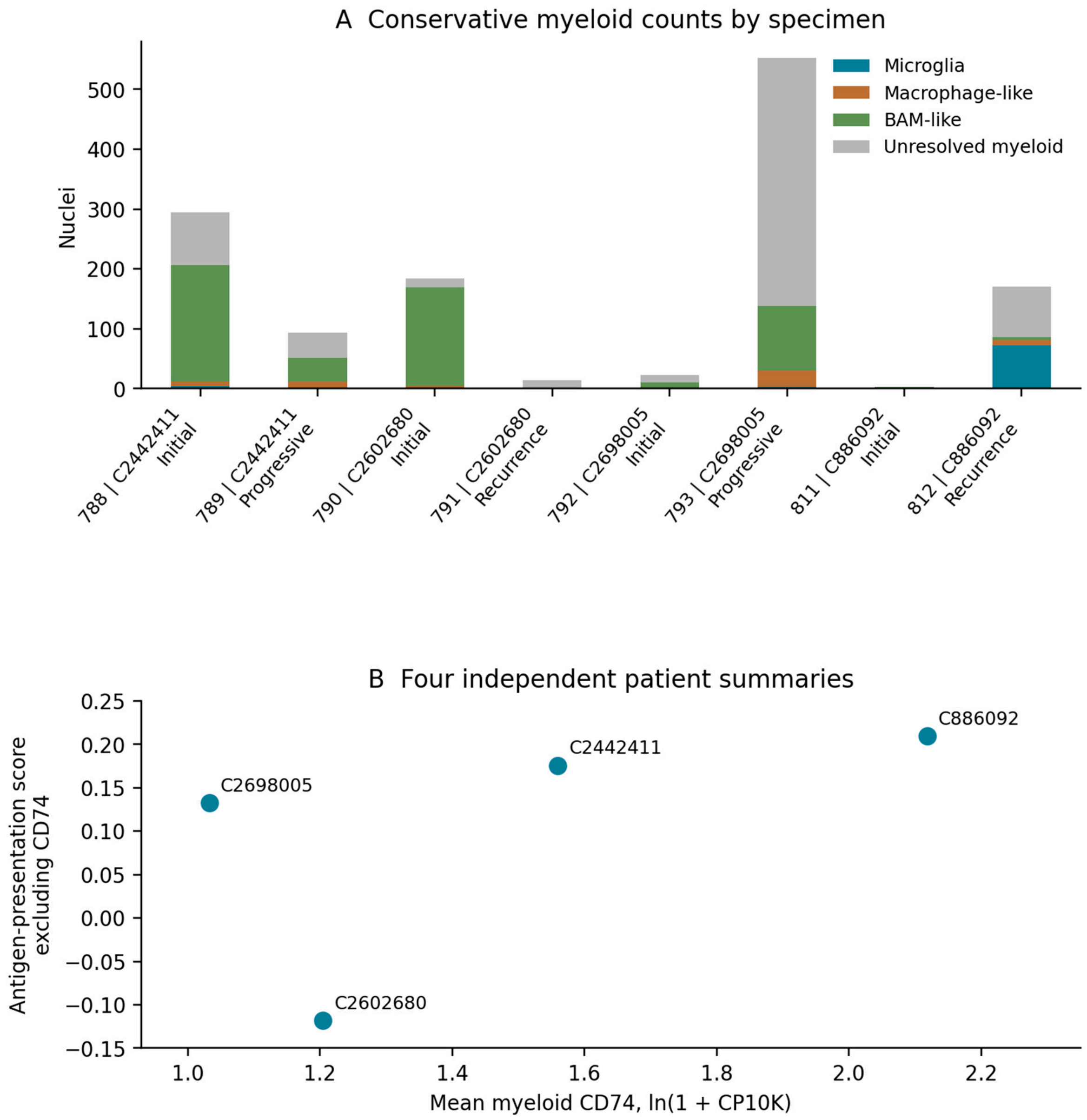


The ScPCA cohort contains 42,703 nuclei in eight specimens from four patients. Source CNV evidence and coherent marker panels define the operational groups. No astrocytes meet the conservative criteria; 72/80 conservative microglia are in SCPCL000812. Patient-level CD74 analysis uses eligible specimen means averaged equally within patient. CD74 is excluded from the antigen-presentation score. The pooled operational myeloid result (rho = 0.800; exact two-sided P = 0.333; BH q = 0.750; four patients) is not statistically significant and is not a microglia-specific validation. Source data and complete tables accompany Table S17.

## Supplementary Figure S8 External CellChat candidates

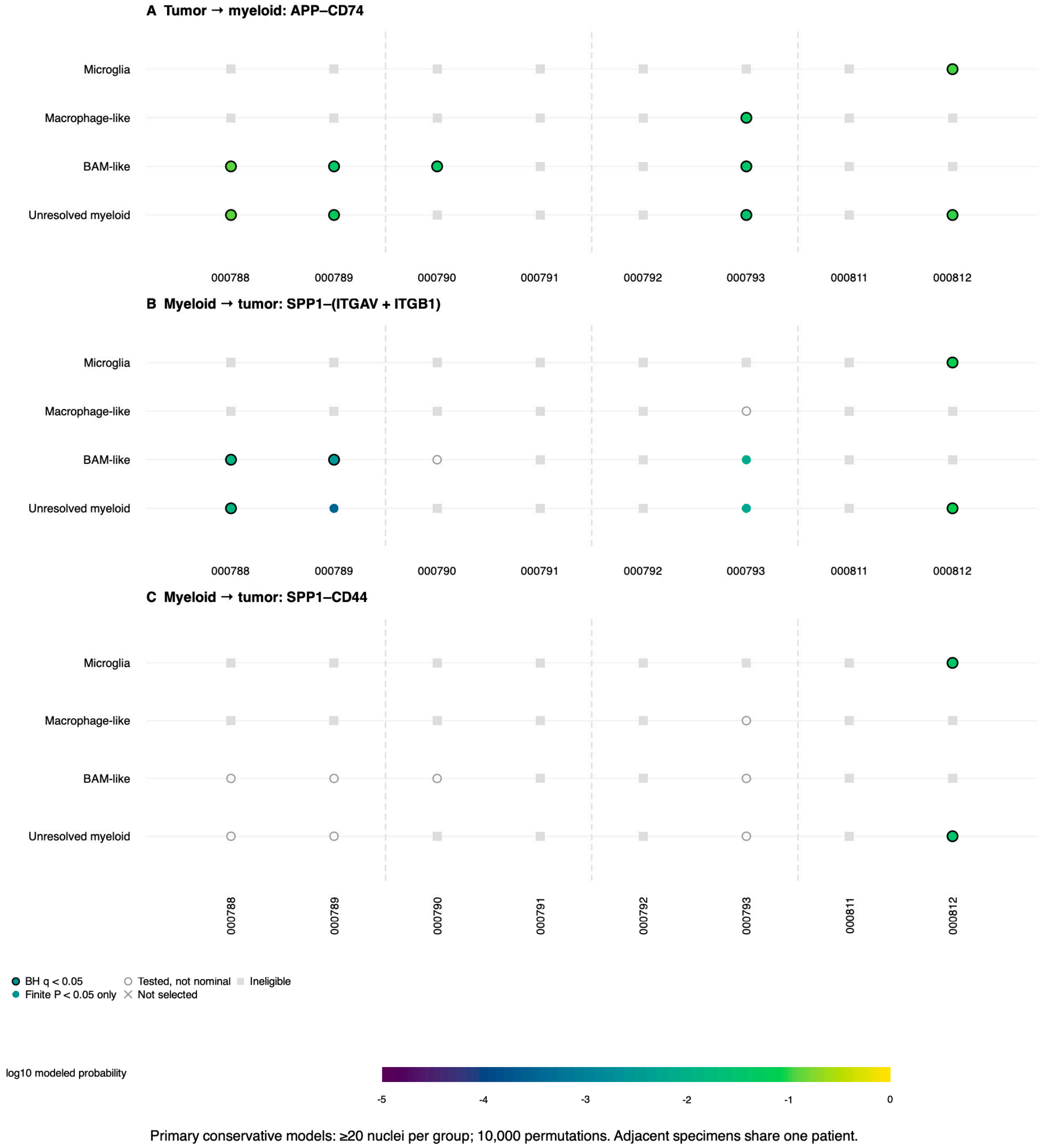


Primary conservative models require at least twenty nuclei per group. (A) Tumor-to-myeloid APP–CD74; (B,C) myeloid-to-tumor SPP1–(ITGAV + ITGB1) and SPP1–CD44. Colored circles with a black outline pass within-model BH correction across all tested entries using finite-permutation P values; colored circles without a black outline meet only finite-permutation P < 0.05. Small gray open circles are tested non-nominal results, crosses denote interactions not selected by the model, and squares denote ineligible groups. Adjacent columns represent paired specimens from the same patient: 788/789, 790/791, 792/793, and 811/812. Source timing and full sample IDs are in Table S14. No inferCNV-supported tumor group was available for 791; 792 and 811 had no eligible myeloid group at twenty. Expression-only tumor candidates were excluded. Color denotes modeled probability for nominally supported results, not a measured signaling rate. Broader-annotation and minimum-ten model results are provided in Table S17; overlapping sensitivity models are not independent observations. All models use 10,000 permutations.

## Supplementary Table S8 Astrocyte threshold sensitivity

| Program | Minimum genes | Positive / total | Percent |
|---|---|---|---|
| ECM-remodeling | 1 | 6 / 47 | 12.8 |
| ECM-remodeling | 2 | 6 / 47 | 12.8 |
| ECM-remodeling | 3 | 6 / 47 | 12.8 |
| Homeostatic | 1 | 12 / 47 | 25.5 |
| Homeostatic | 2 | 12 / 47 | 25.5 |
| Homeostatic | 3 | 12 / 47 | 25.5 |
| Inflammatory | 1 | 5 / 47 | 10.6 |
| Inflammatory | 2 | 5 / 47 | 10.6 |
| Inflammatory | 3 | 5 / 47 | 10.6 |
| Pan-reactive | 1 | 9 / 47 | 19.1 |
| Pan-reactive | 2 | 9 / 47 | 19.1 |
| Pan-reactive | 3 | 9 / 47 | 19.1 |

The original 10,000 matched-null calibrations were retained. Per-cell UCell scores were independently recomputed from raw counts and matched the saved baseline to numerical precision ($<10^{-12}$). All cell-level assignments and overlap patterns are unchanged across k = 1, 2, and 3. The per-cell table retains each cell identifier, sample, subtype, detected-gene count, score, cutoff, and positivity call.

## Supplementary Table S9 Sample inclusion and provenance

| Tumor | All nuclei | TME | Astrocytes | Microglia | State-resolved tumor | All malignant nuclei |
|---|---|---|---|---|---|---|
| MYC-1 | 1058 | 45 | 1 | 31 | 184 | 1013 |
| MYC-2 | 102 | 89 | 4 | 18 | 5 | 13 |
| MYC-3 | 2646 | 58 | 10 | 37 | 2490 | 2588 |
| SHH-1 | 4676 | 66 | 2 | 55 | 928 | 4610 |
| SHH-2 | 2355 | 31 | 3 | 16 | 904 | 2324 |
| SHH-3 | 1033 | 28 | 1 | 22 | 870 | 1005 |
| SHH-4 | 1491 | 56 | 7 | 31 | 1254 | 1435 |
| SHH-5 | 439 | 23 | 2 | 10 | 105 | 416 |
| TYR-1 | 1650 | 48 | 0 | 38 | 1515 | 1602 |
| TYR-2 | 1272 | 33 | 12 | 11 | 292 | 1239 |
| TYR-3 | 168 | 9 | 2 | 5 | 101 | 159 |
| TYR-4 | 674 | 30 | 3 | 14 | 595 | 644 |

All tumor nuclei retain the published Final_Annotation_Focus assignment, including Unannotated and Immune-like malignant nuclei. State-resolved tumor nuclei belong to the eight published malignant states. “Microglia” denotes the published annotation; conservative identity assessment is reported separately. TME counts range from 9 to 89. ATRT-MYC-2 remains in the primary expression and composition summaries but is excluded from the primary CellChat inputs; its inclusion is assessed separately in Table S13. It also fails the donor-specific tumor minimum, contributing only five state-resolved tumor nuclei.

| MYC cohort | TME / all nuclei | TME percent |
|---|---|---|
| All three tumors | 192 / 3,806 | 5.04 |
| Without MYC-2 | 103 / 3,704 | 2.78 |

**Table S9 Continued: subtype tests and data provenance**

| Microglial module | BH q with MYC-2 | BH q without MYC-2 |
|---|---|---|
| Homeostatic | 0.970 | 0.969 |
| Inflammatory | 0.970 | 0.969 |
| Immunoregulatory | 0.663 | 0.969 |
| Interferon-response | 0.970 | 0.969 |
| Phagocytic and lipid processing | 0.970 | 0.969 |
| Antigen-presentation | 0.663 | 0.969 |
| Hypoxia and stress-response | 0.970 | 0.969 |
| Complement | 0.927 | 0.969 |
| Proliferation-associated | 0.970 | 0.969 |

Each test uses donor-mean scores (12 or 11 tumors), the original 288-nucleus standardization scale, a Kruskal–Wallis test, and BH correction over nine modules. Failure to detect a difference does not establish subtype equivalence.

## Provenance verification

The GEO snRNA_10Xv3 series GSE283839 (subseries of GSE283843) was rechecked on 7 October 2026. The record was public on 24 July 2025 and listed a last update of 12 March 2026. The originally reported access date remains 15 February 2026. Both currently deposited downloads exactly match the decompressed local analysis files. Source: https://www.ncbi.nlm.nih.gov/geo/query/acc.cgi?acc=GSE283839

GSE283839_ATRT_RNA_V3_metadata.tsv.gz

Decompressed SHA-256: af6cb000d07fc69a1763701d3c0df9d50e41884031241406821255bc9f1963e2

GSE283839_ATRT_RNA_V3_counts_raw.tsv.gz

Decompressed SHA-256: d2e27ea99c11dc1365cd65ccf8dd1e3fc1e7c04289ffbcdc2f7f98b72fb622fc

## Supplementary Table S10 Microglial gene filter audit

Genes were excluded when detected in fewer than nine of 288 source-labeled microglial nuclei. The manuscript's Table 3 contains the retained genes. The complete machine-readable audit includes every requested gene, detection count, percentage, and retention status.

| Module | Excluded gene | Detected / 288 | Percent |
|---|---|---|---|
| Inflammatory | IL6 | 6 | 2.08 |
| Inflammatory | CXCL9 | 4 | 1.39 |
| Inflammatory | CXCL10 | 5 | 1.74 |
| Inflammatory | CXCL11 | 3 | 1.04 |
| Inflammatory | NOS2 | 1 | 0.35 |
| Immunoregulatory | ARG1 | 8 | 2.78 |
| Immunoregulatory | CCL18 | 4 | 1.39 |
| Immunoregulatory | MARCO | 8 | 2.78 |
| Hypoxia and stress-response | CA9 | 2 | 0.69 |

All requested homeostatic and antigen-presentation genes were retained. CD74 was retained in the original state analysis and removed only from the antigen-presentation score used for the CD74 correlation analysis.

## Supplementary Table S11 CD74 associations

| Module | Spearman rho | Permutation P | BH q |
|---|---|---|---|
| Homeostatic | -0.182 | 0.57213 | 0.66287 |
| Inflammatory | 0.175 | 0.58922 | 0.66287 |
| Immunoregulatory | 0.671 | 0.02048 | 0.09216 |
| Interferon-response | 0.434 | 0.16141 | 0.36502 |
| Phagocytic and lipid processing | 0.182 | 0.57547 | 0.66287 |
| Antigen-presentation | 0.923 | 0.00005 | 0.00045 |
| Hypoxia and stress-response | -0.070 | 0.83403 | 0.83403 |
| Complement | 0.399 | 0.20279 | 0.36502 |
| Proliferation-associated | -0.399 | 0.20014 | 0.36502 |

n = 12 tumors. Correlations use donor means of ln(1 + CP10K) CD74 expression and fixed-scale module scores. CD74 was excluded from the antigen-presentation module to avoid part-whole circularity. Two-sided permutation P values use 99,999 permutations (seed 20261007) and a +1 correction. BH correction is across nine modules. The immunoregulatory association is not significant after this correction.

## Sensitivity and interpretation

Removing MYC-2 retained the antigen-presentation association (rho = 0.909). The nucleus-level partial rank correlation after removing donor indicators and library-depth effects was 0.676 for antigen presentation and 0.426 for the immunoregulatory program; these cell-level summaries are descriptive. The accompanying files also contain donor-level CD74 detection fractions and within-donor correlations. None of these associations demonstrates an APP-induced response, antigen-presentation function, or CD74 activation.

## Supplementary Table S12 Paired astrocyte comparisons

| Module | TYR-2 | MYC-3 | SHH-4 |
|---|---|---|---|
| Cytokines/growth factors | +0.757 | +0.544 | -0.892 |
| ECM/adhesion | +1.212 | -0.277 | -0.864 |
| Lipid/cholesterol | +0.392 | -0.789 | -0.872 |
| Lactate/glucose | -0.679 | -0.742 | -0.894 |
| Glutamate/glutamine | +1.303 | +1.294 | -0.211 |
| Antioxidant/glutathione | -0.698 | -0.615 | -0.154 |
| Extracellular vesicles | -0.469 | -1.076 | -1.036 |
| Organelle transfer | -0.520 | +0.027 | -0.564 |

Values are within-tumor astrocyte-minus-state-resolved-tumor module-score differences, with each score the mean gene-level log2(CPM + 1). TYR-2, MYC-3, and SHH-4 contributed 12, 10, and 7 astrocytes, respectively. The glutamate/glutamine median is +1.294, but SHH-4 has a negative difference. These three-donor observations are descriptive; they do not establish a general increase or measure metabolic flux. Scores omit genes unavailable in the count matrix: the antioxidant/glutathione module uses fourteen of fifteen requested genes because GPX1 is absent. These values reproduce Figure 4A; the machine-readable audit also retains the alternative requested-panel denominator.

## Supplementary Table S13 CellChat sensitivity to tumor inclusion

All models used CellChat 2.1.2 (R 4.4.3), its bundled human database, ln(1 + CP10K) expression, truncatedMean averaging (trim = 0.1), seed 42, and population.size = FALSE. To assess Unannotated inclusion with patient composition and cell numbers controlled, we fitted two tumor definitions separately for MYC, SHH, and TYR: the eight published malignant states and those states plus Unannotated malignant nuclei. Immune-like malignant cells were excluded from both. The restricted models retained the exact original input cells. The expanded models retained the original tumor donors, all astrocyte and source-microglial cell IDs, and the original tumor sampling targets per donor (184 for MYC, 870 for SHH, and 292 for TYR). Tumor nuclei were sampled without replacement from the expanded eligible pool using seed 42 within each donor. The same 3,220 database interactions with complete ligand/receptor gene components represented in the expression matrix were tested in all six models, independently of overexpression screening. All five highlighted candidates were therefore tested under both definitions. Each model used 10,000 permutations and BH adjustment across 28,980 source–target–interaction entries. This is one reproducible balanced sampling comparison, not an exhaustive assessment of sampling variability (Table S13A,C).

CellChat temporarily shuffled cell-group labels across nuclei within each pooled model, preserving group sizes and expression profiles. Saved annotations and donor IDs were unchanged; shuffles were not blocked by donor. Finite-permutation $P = (b + 1)/(B + 1)$, where b is the number of shuffled scores strictly exceeding the observed score; zero observed scores retained $P = 1$. BH adjustment covered every tested source–target–interaction entry within each model. With $B = 10{,}000$, the minimum unadjusted finite-permutation P is 1/10,001. Nominal support denotes finite $P < 0.05$ and BH support denotes $q < 0.05$; native P values are also exported. These within-model tests do not establish differences between molecular subtypes or consistency across independent patients.

**Table S13 Continued: summary of sensitivity analyses**

APP–CD74 passed within-model Benjamini–Hochberg (BH) correction under both definitions in MYC, SHH, and TYR (q = 0.00627–0.00875). For the models including Unannotated nuclei, APP–CD74 q values were MYC 0.00627, SHH 0.00875, TYR 0.0068. The Jaccard overlap of tumor–glia interaction sets between definitions was 0.67–0.76 at finite P < 0.05 and 0.65–0.67 at BH q < 0.05. These results indicate that some candidate associations persist while the broader inferred interaction repertoire depends on tumor inclusion (Table S13A,C). NCAM1–FGFR1 also remained BH-supported in all three subtypes under both definitions. In TYR, SPP1–(ITGAV + ITGB1) and PTN–PTPRZ1 lost BH support after Unannotated inclusion; in MYC, SPP1–CD44 gained BH support. These are changes in within-model support, not statistical tests of changes between definitions.

| Sensitivity comparison | Finding |
|---|---|
| Controlled Unannotated inclusion | APP–CD74 passed within-model Benjamini–Hochberg (BH) correction under both definitions in MYC, SHH, and TYR (q = 0.00627–0.00875). Interaction overlap: 0.67–0.76 at finite P < 0.05; 0.65–0.67 at BH q < 0.05. Patients, group sizes, glial cells and testing family held fixed. |
| All-malignant sensitivity with separate gene screens | Gene-screened all-malignant models retained APP–CD74 after BH correction in MYC and SHH. In TYR, APP did not meet the preliminary gene-screening threshold (P = 0.0833), so the interaction was not tested. This broader tumor definition can change patient eligibility (Table S13B). |
| Restore ATRT-MYC-2 (10,000 permutations) | MYC APP–CD74 probability 0.0532 to 0.0520; within-model BH support retained. Other candidate repertoires changed. |
| Same-donor models (10,000 permutations) | Within-model BH q < 0.05: APP–CD74 7/10; NCAM1–FGFR1 2/3; PTN–PTPRZ1 1/3 eligible tumors. Nominal counts are the same. |
| Conservative populations (10,000 permutations) | Only 1 eligible conservative astrocyte donor and 1 conservative microglial donor at the five-nucleus threshold; insufficient for subtype assessment. |
| Gene-screened models (Table S13B) | With 10,000 permutations, the numbers of state-resolved subtype models with BH support were APP–CD74, 3/3; NCAM1–FGFR1, 3/3; SPP1–(ITGAV + ITGB1), 2/3; SPP1–CD44, 1/3; and PTN–PTPRZ1, 1/3. Historical 100-permutation outputs are retained only in the analysis archive. |

### Table S13A Controlled inclusion of Unannotated tumor nuclei

| Tumor scope | Candidate and direction | MYC q | SHH q | TYR q |
|---|---|---|---|---|
| Eight malignant states | APP–CD74<br>Tumor → microglia | 0.00656 | 0.00771 | 0.00635 |
| Eight malignant states | NCAM1–FGFR1<br>Astrocyte → tumor | 0.00656 | 0.00771 | 0.00635 |
| Eight malignant states | SPP1–(ITGAV + ITGB1)<br>Microglia → tumor | 0.012 | 0.912 | 0.00635 |
| Eight malignant states | SPP1–CD44<br>Microglia → tumor | 0.623 | 1 | 0.0772 |
| Eight malignant states | PTN–PTPRZ1<br>Astrocyte → tumor | 1 | 1 | 0.00635 |
| Eight states plus Unannotated | APP–CD74<br>Tumor → microglia | 0.00627 | 0.00875 | 0.0068 |
| Eight states plus Unannotated | NCAM1–FGFR1<br>Astrocyte → tumor | 0.00627 | 0.0163 | 0.0068 |
| Eight states plus Unannotated | SPP1–(ITGAV + ITGB1)<br>Microglia → tumor | 0.00627 | 1 | 0.101 |
| Eight states plus Unannotated | SPP1–CD44<br>Microglia → tumor | 0.0208 | 1 | 1 |
| Eight states plus Unannotated | PTN–PTPRZ1<br>Astrocyte → tumor | 1 | 1 | 0.654 |

Values are BH-adjusted finite-permutation P values from 10,000 permutations per model (seed 42). All six controlled models test the same 3,220 database interactions and 28,980 source–target–interaction entries; BH adjustment is applied across the full set within each model, not only the five highlighted candidates. The eight malignant states and those states plus Unannotated nuclei use the same patients, group sizes and glial nuclei; Immune-like cells are excluded from both. Every highlighted candidate is tested, including zero-score candidates retained with P = 1. These q-values use a common testing family and are reported separately from the gene-screened models in Table S13B. Full scores, exceedance counts, P values, q-values and input cell IDs accompany the table.

The accelerated calculation preserves native CellChat permutation indices, group sizes, score equations, receptor complexes, cofactors and the strict exceedance rule. Seven numerical-equivalence checks are archived. A 37-permutation comparison (seed 271828) of the complete 3,220-interaction family in the controlled MYC-plus-Unannotated input matched unmodified CellChat exactly for every observed score and permutation P value. For each controlled restricted model, all entries shared with its original 10,000-permutation model retained exactly the same observed score and native permutation P. All controlled BH values were independently reproduced. Code, saved objects, exact input IDs, donor contributions and checksums accompany Table S13.

## Table S13B Tumor definitions evaluated with separate gene screens

Six subtype models compared the eight published malignant states with all published malignant populations meeting the group thresholds, including Unannotated and Immune-like nuclei. Eligibility and sampling counts could therefore differ between definitions. These models used separate gene-selected interaction families and 10,000 permutations; the state-resolved models provide the results in Figure 6A,B. MYC-2 inclusion, donor-specific, and conservative-population models also used 10,000 permutations. Donor-specific models required at least 20 state-resolved tumor nuclei and, for the relevant interaction, at least 10 microglial or five astrocyte nuclei. The pooled MYC-2 inclusion sensitivity added its 18 microglial nuclei and changed microglial downsampling; its tumor and astrocyte populations remained ineligible. The computational checks preserved each saved model's input cells, interaction-selection rules, and observed scores.

| Tumor scope | Candidate and direction | MYC q | SHH q | TYR q |
|---|---|---|---|---|
| Eight malignant states | APP–CD74<br>Tumor → microglia | 0.0026 | 0.00284 | 0.00194 |
| Eight malignant states | NCAM1–FGFR1<br>Astrocyte → tumor | 0.0026 | 0.00284 | 0.00194 |
| Eight malignant states | SPP1–(ITGAV + ITGB1)<br>Microglia → tumor | 0.00484 | 0.37 | 0.00194 |
| Eight malignant states | SPP1–CD44<br>Microglia → tumor | 0.289 | 0.508 | 0.0253 |
| Eight malignant states | PTN–PTPRZ1<br>Astrocyte → tumor | 1 | 1 | 0.00194 |
| All published malignant | APP–CD74<br>Tumor → microglia | 0.00219 | 0.00323 | Not tested* |
| All published malignant | NCAM1–FGFR1<br>Astrocyte → tumor | 0.00219 | 0.0102 | 0.00212 |
| All published malignant | SPP1–(ITGAV + ITGB1)<br>Microglia → tumor | 0.00219 | 0.0552 | 0.0305 |
| All published malignant | SPP1–CD44<br>Microglia → tumor | 0.00219 | 0.242 | 1 |
| All published malignant | PTN–PTPRZ1<br>Astrocyte → tumor | 1 | 1 | 1 |

* In the gene-screened all-malignant TYR analysis using CellChat's overexpression screen, APP had a tumor-versus-other-groups Wilcoxon $P = 0.0833$, above the unadjusted $P < 0.05$ screening threshold; CD74 passed in source-labeled microglia. With positive log fold change required and both ligand and receptor required in the selected gene list, APP–CD74 was excluded before interaction testing, so no interaction P or q was calculated. APP passed the same screen in the gene-screened state-resolved TYR model ($P = 0.0101$). This screening outcome does not establish biological absence. The controlled comparison tests a common database interaction set and therefore provides an APP–CD74 result under both definitions. The original interaction testing families differed between models; the q-values shown use 10,000 permutations and within-model correction. Nominal (native $P < 0.05$) and BH-supported repertoire overlaps were 0.51–0.56 and 0.49–0.50, respectively. The paired controlled comparison in Table S13A addresses Unannotated inclusion directly.

### Table S13C Fixed tumor sampling and patient contributions

| Subtype | Tumor donors | Nuclei per donor | Total tumor nuclei | Unannotated in expanded sample |
|---|---|---|---|---|
| MYC | MYC-1, MYC-3 | 184 | 368 | 166 |
| SHH | SHH-1, SHH-2, SHH-3, SHH-4 | 870 | 3480 | 1353 |
| TYR | TYR-1, TYR-2, TYR-4 | 292 | 876 | 251 |

Tumor counts and donor membership are identical under both controlled definitions. The original source-label glial inputs are unchanged: MYC, 10 astrocyte and 62 microglial nuclei; SHH, 7 and 50; TYR, 12 and 33. Glial donor contributions may differ from tumor contributions but are identical between definitions. No Immune-like cells are included. Expanded sampling uses seed 42 within each donor; counts of Unannotated nuclei are realized sample counts, not the full eligible pool. Exact cell IDs, per-donor counts, the analysis plan, gene-screen audit, and full numerical results are retained in the accompanying controlled analysis package.

## Reproducibility and scope

The analysis package contains saved CellChat objects, exact input cell identifiers, donor contributions, database interaction and complex definitions, native permutation outputs, finite-permutation P values, and within-model BH values. All malignant labels retain their published assignments. The pooled astrocyte display in Figure 4D is retained from the initial analysis, for which the original CellChat objects and exact database snapshot were unavailable. Figure 6 uses the documented CellChat 2.1.2 models with 10,000 permutations (Table S13B). Subtype comparisons are descriptive. Donor-specific models assess candidates within individual tumors, with eligibility determined by the available group sizes.

## Additional conservative population fits

Conservative annotation retains 23 of the source-labeled astrocytes and 15 of the source-labeled microglial nuclei. With a five-nucleus glial minimum, one donor was eligible for an astrocyte network and one for a microglial network; these donor numbers do not permit molecular-subtype comparisons. No donor met a ten-nucleus conservative microglial minimum. Macrophage-like and BAM-like populations were analyzed separately as expression-defined groups. Published malignant assignments were retained throughout.

These additional fits use previously frozen conservative TME identity assignments, not new malignant annotations. Their full marker rules and cell registries accompany the analysis package. Macrophage/BAM-like labels are expression phenotypes and do not prove infiltrating or anatomical border origin. These exploratory fits are not independent validation and are reported separately from the primary source-label analyses.

## Supplementary Table S14 Clinical metadata

The analyzed cohort is the 12-tumor 10X v3 subset of the larger 19-tumor atlas. The published supplement reports snap-frozen tissue for all 12 and extended labels SHH-1A for SHH-1 through SHH-4 and SHH-2 for SHH-5; broad labels are MYC (3), SHH (5), and TYR (4). Age, tumor grade, site, treatment, and outcomes are not reported for these 12 tumors in the deposited GEO metadata or the relevant published patient table. The published per-nucleus gender field is missing for all 12; sex values for the seven other atlas tumors were not transferred to this subset. Table S14 lists source identifiers, reported tissue and technology, and explicit missing fields. The 17,564 cell identifiers and malignant/TME assignments match the published supplement exactly.

| Tumor | Source identifier | Extended subgroup | Nuclei |
|---|---|---|---|
| MYC-1 | H049-ATRT-0001 | MYC | 1058 |
| MYC-2 | H049-JVCT | MYC | 102 |
| MYC-3 | H049-UV6K | MYC | 2646 |
| SHH-1 | H049-ATRT-0005 | SHH-1A | 4676 |
| SHH-2 | H049-ATRT-0007 | SHH-1A | 2355 |
| SHH-3 | H049-ATRT-0009 | SHH-1A | 1033 |
| SHH-4 | H049-ATRT-0014 | SHH-1A | 1491 |
| SHH-5 | H049-ATRT-0022-P | SHH-2 | 439 |
| TYR-1 | H049-ATRT-0003 | TYR | 1650 |
| TYR-2 | H049-ATRT-0006 | TYR | 1272 |
| TYR-3 | H049-ATRT-0010 | TYR | 168 |
| TYR-4 | H049-ATRT-0013 | TYR | 674 |

All 12 were reported as snap frozen. Technology is preserved verbatim as “10X v3 5'” in the machine-readable table. Age, sex, grade, site, treatment, and outcome are explicitly marked not reported; no values were inferred from disease conventions or other patients. The source supplement archive was verified against its published MD5 checksum, and all 17,564 cell IDs, donor assignments, and malignant/TME labels matched exactly.

## External ScPCA clinical information

| Library ID* | Patient | Sex | Timing | Age years and timing |
|---|---|---|---|---|
| 000788 | C2442411 | F | Initial | 3.3 at diagnosis |
| 000789 | C2442411 | F | Progressive | 4.42 at collection |
| 000790 | C2602680 | M | Initial | 4.48 at diagnosis |
| 000791 | C2602680 | M | Recurrence | 5.3 at collection |
| 000792 | C2698005 | F | Initial | 0.95 at diagnosis |
| 000793 | C2698005 | F | Progressive | 2.66 at collection |
| 000811 | C886092 | F | Initial | 0.99 at diagnosis |
| 000812 | C886092 | F | Recurrence | 1.81 at collection |

* Library IDs omit the common SCPCL prefix (for example, 000788 denotes SCPCL000788). The full supplied table retains specimen IDs, tissue site, treatment fields, clinical status, sequencing provenance, and ontology identifiers. Disease timing defines patient_group and is not a molecular subgroup. MYC/SHH/TYR and tumor grade were not supplied. Four paired patients are the independent units; eight libraries are not eight independent patients. No clinical-outcome association was attempted.

## Supplementary Table S15 Annotation and scoring sensitivities

### Table S15A Astrocyte annotation sensitivity

| Astrocyte definition | Nuclei | Eligible paired donors |
|---|---|---|
| Source label | 47 | 3 |
| Conservative source intersection | 23 | 1 |
| Expanded within source TME | 28 | 1 (TYR-2) |
| Non-transporter identity | 14 | 0 |

The original astrocyte state-program scores and 10,000 matched-null cutoffs were held fixed for source-cell subsets. Conservative calls at the two-gene safeguard were homeostatic 12/23, pan-reactive 5/23, inflammatory 3/23, and ECM-associated 5/23. TYR-2 was the only conservative paired donor (six astrocytes; glutamate-module difference +1.326 log2(CPM + 1)). Non-transporter identity produced no eligible paired donor. These selected-set percentages do not estimate all astrocyte states.

Microglial donor counts and eligibility are shown in Table S15B, source-microglial scoring comparisons in Table S15C, and all 75 single-gene omission runs in Table S15D. Source-microglial subtype tests remained non-significant under rank scoring (minimum BH $q = 0.621$) and removal of the detection filter (minimum BH $q = 0.562$). Conservative microglia had only one donor with at least five nuclei, precluding subtype tests. Single-gene omission changed the highest-relative-score display label for 2–18 of 288 nuclei (0.7–6.3%); this does not validate the labels as functional states.

## Table S15B Microglial nucleus counts and donor eligibility

Source-labeled microglia are the 288 nuclei assigned to this compartment in the published annotation. The conservative subset contains 15 nuclei. Eligibility for donor-level scoring requires at least five relevant nuclei; subtype comparisons additionally require at least two eligible donors in each of MYC, SHH, and TYR. These scoring thresholds are separate from CellChat eligibility thresholds.

| Patient | Source microglial nuclei | Conservative microglial nuclei | Source donor eligible | Conservative donor eligible |
|---|---|---|---|---|
| MYC-1 | 31 | 0 | Yes | No |
| MYC-2 | 18 | 0 | Yes | No |
| MYC-3 | 37 | 8 | Yes | Yes |
| SHH-1 | 55 | 0 | Yes | No |
| SHH-2 | 16 | 1 | Yes | No |
| SHH-3 | 22 | 2 | Yes | No |
| SHH-4 | 31 | 4 | Yes | No |
| SHH-5 | 10 | 0 | Yes | No |
| TYR-1 | 38 | 0 | Yes | No |
| TYR-2 | 11 | 0 | Yes | No |
| TYR-3 | 5 | 0 | Yes | No |
| TYR-4 | 14 | 0 | Yes | No |
| Total / eligible donors | 288 | 15 | 12 donors | 1 donor |

All 12 source-microglial donors qualify: MYC, 3; SHH, 5; TYR, 4. Only MYC-3 qualifies for the conservative subset (eight nuclei), so conservative subtype comparisons are not assessable. Original source labels and malignant assignments are unchanged. Full donor means under all three scoring methods are supplied in S16_myeloid_scores_by_donor.csv.

## Table S15C Source microglial subtype tests across scoring methods

Each entry shows the donor-level Kruskal–Wallis P value followed by its BH-adjusted q-value (P / q). Tests compare MYC, SHH, and TYR using 12 donor means (3, 5, and 4 donors, respectively). BH correction covers nine programs separately for each scoring method. All q-values exceed 0.05; this does not establish equivalence between subtypes.

| Program | Baseline z-score P / BH q | Rank score P / BH q | No detection filter P / BH q |
|---|---|---|---|
| Homeostatic | 0.660 / 0.970 | 0.695 / 0.908 | 0.660 / 0.910 |
| Inflammatory | 0.970 / 0.970 | 0.985 / 0.985 | 0.707 / 0.910 |
| Immunoregulatory | 0.090 / 0.663 | 0.069 / 0.621 | 0.062 / 0.562 |
| Interferon-response | 0.880 / 0.970 | 0.807 / 0.908 | 0.880 / 0.933 |
| Phagocytic and lipid processing | 0.572 / 0.970 | 0.731 / 0.908 | 0.572 / 0.910 |
| Antigen-presentation | 0.147 / 0.663 | 0.338 / 0.908 | 0.147 / 0.663 |
| Hypoxia and stress-response | 0.605 / 0.970 | 0.538 / 0.908 | 0.605 / 0.910 |
| Complement | 0.309 / 0.927 | 0.559 / 0.908 | 0.309 / 0.910 |
| Proliferation-associated | 0.933 / 0.970 | 0.604 / 0.908 | 0.933 / 0.933 |

Baseline scores are unweighted means of gene-wise z-scores from ln(1 + CP10K) expression, standardized across the 288 source-labeled microglial nuclei with sample standard deviations. Genes detected in fewer than nine nuclei are omitted. Rank scores use the same retained genes and within-cell ranks capped at 1,500. The no-detection-filter analysis includes all available genes in each original program while retaining the same standardization scale. Donor scores are means of the corresponding cell scores. Complete unrounded values and results for other expression-defined myeloid groups are retained in S16_myeloid_scoring_tests.csv; donor means are in S16_myeloid_scores_by_donor.csv.

## Table S15D Single-gene omission and microglial display labels

Each row is one sensitivity run in the same 288 source-labeled microglial nuclei. One retained gene was omitted from the indicated program, only that program score was recalculated on the fixed baseline standardization scale, and the highest-relative-score label was reassigned using the original program order to resolve ties. The table reports nuclei whose display label changed relative to baseline. No nuclei were removed. Multiple programs can coexist; these labels do not establish a dominant biological function.

The 75 gene–program entries represent 73 distinct genes because TREM2 and STAT1 each contribute to two programs. A shared gene was omitted from one program at a time. The number of changed labels ranged from 2 to 18 of 288 nuclei (0.7–6.3%, rounded). Individual percentages below are shown to two decimal places.

| Program | Omitted gene | Nuclei with changed label | Percent changed |
|---|---|---|---|
| Homeostatic | P2RY12 | 8 / 288 | 2.78 |
| Homeostatic | TMEM119 | 10 / 288 | 3.47 |
| Homeostatic | SALL1 | 8 / 288 | 2.78 |
| Homeostatic | HEXB | 8 / 288 | 2.78 |
| Homeostatic | CX3CR1 | 8 / 288 | 2.78 |
| Homeostatic | GPR34 | 5 / 288 | 1.74 |
| Homeostatic | SLC2A5 | 6 / 288 | 2.08 |
| Homeostatic | BIN1 | 5 / 288 | 1.74 |
| Homeostatic | CSF1R | 7 / 288 | 2.43 |
| Homeostatic | TREM2 | 6 / 288 | 2.08 |
| Inflammatory | IL1B | 9 / 288 | 3.13 |
| Inflammatory | TNF | 4 / 288 | 1.39 |
| Inflammatory | CCL2 | 7 / 288 | 2.43 |
| Inflammatory | CCL3 | 2 / 288 | 0.69 |
| Inflammatory | CCL4 | 4 / 288 | 1.39 |
| Inflammatory | NFKBIA | 6 / 288 | 2.08 |
| Inflammatory | STAT1 | 3 / 288 | 1.04 |
| Inflammatory | IRF1 | 7 / 288 | 2.43 |
| Inflammatory | CD86 | 4 / 288 | 1.39 |
| Immunoregulatory | MRC1 | 13 / 288 | 4.51 |
| Immunoregulatory | CD163 | 9 / 288 | 3.13 |
| Immunoregulatory | MSR1 | 7 / 288 | 2.43 |
| Immunoregulatory | IL10 | 6 / 288 | 2.08 |
| Immunoregulatory | TGFB1 | 15 / 288 | 5.21 |
| Immunoregulatory | MAFB | 10 / 288 | 3.47 |
| Immunoregulatory | VSIG4 | 9 / 288 | 3.13 |
| Immunoregulatory | FOLR2 | 11 / 288 | 3.82 |

| Program | Omitted gene | Nuclei with changed label | Percent changed |
|---|---|---|---|
| Interferon-response | ISG15 | 9 / 288 | 3.13 |
| Interferon-response | IFIT1 | 9 / 288 | 3.13 |
| Interferon-response | IFIT2 | 8 / 288 | 2.78 |
| Interferon-response | IFIT3 | 3 / 288 | 1.04 |
| Interferon-response | MX1 | 8 / 288 | 2.78 |
| Interferon-response | OAS1 | 9 / 288 | 3.13 |
| Interferon-response | OAS2 | 8 / 288 | 2.78 |
| Interferon-response | GBP1 | 9 / 288 | 3.13 |
| Interferon-response | STAT1 | 7 / 288 | 2.43 |
| Interferon-response | IRF7 | 9 / 288 | 3.13 |
| Phagocytic and lipid processing | APOE | 9 / 288 | 3.13 |
| Phagocytic and lipid processing | APOC1 | 7 / 288 | 2.43 |
| Phagocytic and lipid processing | LPL | 9 / 288 | 3.13 |
| Phagocytic and lipid processing | SPP1 | 6 / 288 | 2.08 |
| Phagocytic and lipid processing | GPNMB | 13 / 288 | 4.51 |
| Phagocytic and lipid processing | TREM2 | 10 / 288 | 3.47 |
| Phagocytic and lipid processing | CTSD | 6 / 288 | 2.08 |
| Phagocytic and lipid processing | CTSB | 5 / 288 | 1.74 |
| Phagocytic and lipid processing | LGALS3 | 9 / 288 | 3.13 |
| Phagocytic and lipid processing | FABP5 | 10 / 288 | 3.47 |
| Phagocytic and lipid processing | ABCA1 | 8 / 288 | 2.78 |
| Antigen-presentation | HLA-DRA | 8 / 288 | 2.78 |
| Antigen-presentation | HLA-DRB1 | 7 / 288 | 2.43 |
| Antigen-presentation | HLA-DPA1 | 6 / 288 | 2.08 |
| Antigen-presentation | HLA-DPB1 | 8 / 288 | 2.78 |
| Antigen-presentation | CD74 | 7 / 288 | 2.43 |
| Antigen-presentation | CIITA | 8 / 288 | 2.78 |
| Antigen-presentation | B2M | 5 / 288 | 1.74 |
| Antigen-presentation | TAP1 | 10 / 288 | 3.47 |
| Hypoxia and stress-response | HIF1A | 7 / 288 | 2.43 |
| Hypoxia and stress-response | VEGFA | 9 / 288 | 3.13 |
| Hypoxia and stress-response | LDHA | 8 / 288 | 2.78 |
| Hypoxia and stress-response | BNIP3 | 11 / 288 | 3.82 |
| Hypoxia and stress-response | NDRG1 | 11 / 288 | 3.82 |
| Hypoxia and stress-response | SLC2A1 | 15 / 288 | 5.21 |
| Hypoxia and stress-response | HMOX1 | 5 / 288 | 1.74 |

| Program | Omitted gene | Nuclei with changed label | Percent changed |
|---|---|---|---|
| Complement | C1QA | 16 / 288 | 5.56 |
| Complement | C1QB | 12 / 288 | 4.17 |
| Complement | C1QC | 15 / 288 | 5.21 |
| Complement | C3 | 12 / 288 | 4.17 |
| Complement | CFB | 13 / 288 | 4.51 |
| Complement | SERPING1 | 18 / 288 | 6.25 |
| Proliferation-associated | MKI67 | 7 / 288 | 2.43 |
| Proliferation-associated | TOP2A | 11 / 288 | 3.82 |
| Proliferation-associated | CENPF | 12 / 288 | 4.17 |
| Proliferation-associated | UBE2C | 9 / 288 | 3.13 |
| Proliferation-associated | TYMS | 8 / 288 | 2.78 |
| Proliferation-associated | PCNA | 8 / 288 | 2.78 |

The complete machine-readable omission results are supplied in S16_largest_relative_score_LOO.csv. The data package accompanying Table S15 also includes astrocyte results, all myeloid scoring tests, donor means, and explicit donor eligibility.

## Supplementary Table S16 Donor support and receptor definition

| Population and candidate | Minimum TME nuclei | Eligible donors | Finite P < 0.05 / BH q < 0.05 |
|---|---|---|---|
| Source astrocytes NCAM1–FGFR1 | 5 | 3 | 2 / 2 |
| Source astrocytes NCAM1–FGFR1 | 10 | 2 | 1 / 1 |
| Source astrocytes PTN–PTPRZ1 | 5 | 3 | 1 / 1 |
| Source astrocytes PTN–PTPRZ1 | 10 | 2 | 1 / 1 |
| Source astrocytes either candidate | 20 | 0 | Not assessable |
| Conservative astrocytes either candidate | 10 | 0 | Not assessable |
| Source microglial APP–CD74 | 20 | 6 | 5 / 5 |

Higher cell-count requirements were applied as eligibility filters to fixed donor networks; overexpression selection and networks were not refitted. The tumor minimum remained 20 nuclei. Primary donor models required at least 10 source-labeled microglial nuclei or five astrocyte nuclei for the relevant interaction. BH correction retained each model's complete testing family. Source data distinguish ineligible groups, interactions excluded by the gene screen, tested interactions without nominal support, and interactions with nominal or BH support. Expression tables report positive-cell counts, detection fractions, normalized means, and simultaneous detection of all receptor subunits. Figure 6 uses exact database receptor definitions, including SPP1–(ITGAV + ITGB1). All reported donor networks use 10,000 permutations.

## Supplementary Table S17 External cohort analyses

Primary annotation retained 27,572 CNV-supported tumor candidates, 80 microglia, 60 macrophage-like, 525 BAM-like, and 671 unresolved myeloid cells. No astrocytes met the conservative criteria. The broader tumor set contained 30,375 nuclei. Source classifier labels are supporting evidence and were not treated as interchangeable ground truth. The no-inferCNV library SCPCL000791 has expression-only candidates that remain excluded from tumor models. Operational RNA-derived CNV is not DNA confirmation.

Of 24 prespecified external model configurations, 17 eligible configurations were evaluated (16 distinct input cell sets) and 7 were skipped because fewer than two groups or no CNV-supported tumor group met eligibility. In the primary conservative models (minimum 20 nuclei per group), tumor-to-myeloid APP–CD74 passed within-model BH correction in at least one eligible myeloid population in 5/5 evaluable specimens, representing 4/4 evaluable patients; myeloid-to-tumor SPP1–(ITGAV + ITGB1) passed in 3/5 evaluable specimens, representing 2/4 evaluable patients; and myeloid-to-tumor SPP1–CD44 passed in 1/5 evaluable specimens, representing 1/4 evaluable patients. All models used 10,000 permutations. A patient is counted once when at least one specimen supports a candidate. Broader-annotation and minimum-ten sensitivities are reported with all eligible denominators in Table S17. These counts summarize recurrence of within-model support, not a patient-level significance test. Astrocyte candidates were not assessable. Repeated computational support does not establish receptor activation or confirm a microglia-specific mechanism (Figure S8; Table S17).

| Library ID* | Patient | Conservative 20 | Broader 20 | Conservative 10 |
|---|---|---|---|---|
| 000788 | C2442411 | Complete | Complete | Complete |
| 000789 | C2442411 | Complete | Complete | Complete |
| 000790 | C2602680 | Complete | Complete | Complete |
| 000791 | C2602680 | Skipped | Skipped | Skipped |
| 000792 | C2698005 | Skipped | Skipped | Complete |
| 000793 | C2698005 | Complete | Complete | Complete |
| 000811 | C886092 | Skipped | Skipped | Complete |
| 000812 | C886092 | Complete | Complete | Complete |

**Table S17 Continued: model settings and patient-level summaries**

* Library IDs omit the common SCPCL prefix. All external models use CellChat 2.1.2 and its full human database, ln(1 + CP10K) based on full-library counts, truncatedMean with trim 0.1, population.size = FALSE, 10,000 permutations, and seed 20261008. Tables include every tested source–target–interaction entry, native P, finite-permutation P = (b + 1)/10,001, and BH q across the complete within-model family. Zero observed scores retain P = 1. Nominal support uses finite P < 0.05. Inclusive group thresholds are implemented with internal min.cells one below the stated minimum. All 17 eligible configurations were recomputed with unchanged input cells and groups; acceleration was checked against every saved original model, and full-family BH values were independently reproduced. A patient counts once if any eligible specimen supports the candidate; recurrence is descriptive and is not a patient-level significance test. Broad and conservative models overlap and do not supply additional independent patients. The conservative ten- and twenty-cell configurations for SCPCL000793 contain identical nuclei and groups and produced identical networks when recomputed, yielding sixteen distinct input cell sets across seventeen eligible configurations.

CD74 association testing uses four independent patient summaries, with equal weighting of eligible specimens within patient and CD74 excluded from correlated modules. The antigen-presentation result is rho = 0.800, exact two-sided P = 0.333, BH q = 0.750. BAM-like cells support three patient summaries; microglia and macrophage-like groups each support only one. No program association passed BH correction. With four patients, even perfect rank correlation has exact two-sided P = 2/24 = 0.083; the available sample size cannot achieve P < 0.05 and the result is not evidence against the discovery association. Exact cell IDs, sample and patient labels, annotation tiers, source-method comparisons, CNV thresholds, expression data, and full sensitivity denominators are included.

## Supplementary Table S18 Cell2Sentence implementation audit

| Setting | Value |
|---|---|
| Model | vandijklab/C2S-Scale-Gemma-2-27B |
| Organism | Homo sapiens |
| Gene ordering | Up to 1,000 genes by decreasing expression; raw-count ranks |
| Input token limit | 4,096 |
| Output token limit | 24 |
| Generation | Greedy; do_sample=False |
| Seed | 42 |
| Batch size | 8 |
| Quantization | 4-bit NF4; double quantization; bfloat16 compute |
| Padding | Left |
| Cell inclusion | All 516 source TME nuclei; no confidence cutoff |
| Label harmonization | Eight broad categories; mapping function archived |
| Exact TME execution log | Not retained |
| Immutable model revision | Not recorded |
| Installed package versions | Not pinned in supplied template |
| Input paths in template | Example tumor-cell paths remain in original template |
| Interpretation | Same-data method comparison; not independent validation |

The supplied template asks the model to assign a cell type from 1,000 genes ordered by decreasing expression in a Homo sapiens cell. The exact literal prompt, code hashes, and all 516 saved cell sentences and predicted labels are archived. The template does not establish the exact historical TME invocation: input-path placeholders, unpinned dependencies, a missing immutable model revision, and missing execution logs remain documented gaps. No retrospective confidence cutoff was imposed. Broad-lineage agreement of 72.9% with GEO is a same-dataset annotation comparison, not biological validation.

## File organization and reproducibility

Tables S1–S7 are supplied separately and retain the original cohort, gene-set, candidate, annotation-comparison, and abbreviation information, with updated labels and reference numbering. Archived filenames retain their original prefixes: S15 files accompany main-text Table 1, and S16–S19 files accompany current Tables S15–S18, respectively. Other table prefixes are unchanged. The accompanying analysis package includes cell registries, patient and specimen labels, figure source data, code, provenance, and model outputs. Its README describes execution order and the locations of required input files.
